# Melt Electrowriting of Elastic Scaffolds Using PEOT‐PBT Multi‐block Copolymer

**DOI:** 10.1002/adhm.202402914

**Published:** 2024-12-10

**Authors:** Armin Amirsadeghi, Pavan Kumar Reddy Gudeti, Sietse Tock, Marcus Koch, Daniele Parisi, Marleen Kamperman, Małgorzata Katarzyna Włodarczyk‐Biegun

**Affiliations:** ^1^ Polymer Science Zernike Institute for Advanced Materials University of Groningen Nijenborgh 3 Groningen 9747 AG The Netherlands; ^2^ Biotechnology Centre The Silesian University of Technology B. Krzywoustego 8 Gliwice 44‐100 Poland; ^3^ INM – Leibniz Institute for New Materials Campus D2 2 66123 Saarbrücken Germany; ^4^ Engineering and Technology Institute Groningen (ENTEG) University of Groningen Nijenborgh 3 Groningen 9747 AG The Netherlands

**Keywords:** 3D printing, fibroblasts, melt electrowritten scaffolds, thermoplastic elastomer, tissue engineering

## Abstract

Melt electrowriting (MEW) is a powerful additive manufacturing technique to produce tissue engineering scaffolds. Despite its strength, it is limited by a small number of processable polymers. Therefore, to broaden the library of materials for MEW, we investigated the printability of poly(ethylene oxide terephthalate)‐poly(butylene terephthalate) (PEOT‐PBT), a thermoplastic elastomer. The effect of different printing parameters and material thermal degradation are studied. It is observed that the material is stable for >60 min at a printing temperature of 195 °C in a nitrogen environment. Next, two types of designs are printed and characterized: mesh‐like and semi‐random scaffolds. For both types of designs, PEOT‐PBT scaffolds reveal a higher yield strain, and lower Young's modulus as compared to control polycaprolactone scaffolds. Biological studies performed using mouse embryonic fibroblasts (NIH‐3T3) show good cell viability and metabolic activity on all print scaffolds. SEM imaging reveals actively migrating cells on PEOT‐PBT mesh scaffolds after 24 h of culture and 98.87% of pore bridging by cells after 28 days of culture. Immunofluorescence staining shows decreased expression of alpha‐smooth muscle actin from day 14 to day 28 in PEOT‐PBT mesh scaffolds. Overall, it is shown that melt electrowritten PEOT‐PBT scaffolds have great potential for soft tissue regeneration.

## Graphical abstract

This study expands the material choices for melt electrowriting by exploring the printability of PEOT‐PBT, a thermoplastic elastomer. Various physicochemical characterizations are performed to study the material's properties before and after printing. Through optimized printing parameters, stable, soft tissue‐compatible scaffolds are produced. The resulting PEOT‐PBT scaffolds support fibroblast viability and reduce α‐SMA expression, offering the potential for tissue engineering applications.

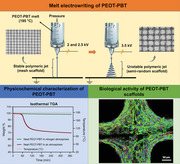

## 1 Introduction

A natural extracellular matrix (ECM) is a fibrous structure that acts as a support for cell attachment, growth, and migration. ECM also gives the native tissue its unique mechanical properties and structural integrity.^[^ ^1^, ^2^, ^3^ ^]^ Most tissue injuries and diseases are associated with damage to cells and the surrounding ECM. Providing external support for cell attachment and growth can facilitate the healing process and is a typical approach in scaffold‐based tissue engineering and regenerative medicine.^[^ ^4^ ^]^

In recent decades, additive manufacturing techniques gained a lot of interest for building tissue‐ and patient‐specific scaffolds for tissue reconstruction due to the high control over micro‐and macro‐structure of fabricated constructs and the possibility to process different materials, tailored to the needs.^[^ ^5^ ^]^ Such approaches as extrusion (bio)printing of soft materials, fused deposition modeling (FDM), electrospinning, and melt electrowriting (MEW) of natural and synthetic polymeric materials, were successfully implemented.^[^ ^6^, ^7^ ^]^ The dimensional precision of these techniques varies from a few nanometers to hundreds of micrometers.^[^ ^8^ ^]^ While FDM and 3D extrusion (bio)printing result in relatively thick fibers (micrometer to millimeter range) electrospinning is able to produce fibers in the nanometer range. However, in the latter one, the control over fiber deposition and the overall shape of the printed construct is limited.^[^ ^9^ ^]^

MEW offers the combined advantages of 3D printing and electrospinning. This method enables direct material deposition known for extrusion and FDM printing, however, with increased printing resolution and smaller fiber diameters (typically 5‐ 20 µm). This fiber size is relevant for biomedical applications, staying within the close range of myofibrils or tendon sub‐fascicles, and can facilitate regenerating ECM.^[^ ^10^ ^]^ Typically, the distance between deposited fibers is well‐controlled, down to 100 µm (40 µm‐ the lowest reported fiber‐to‐fiber distance^[^ ^11^ ^]^), allowing good cell infiltration and easier production of volumetric structures when compared to electrospinning. In MEW, the polymer melt is deposited on the collector plate by applying air pressure in the presence of an electrical field. Fiber‐by‐fiber stacking using the controlled movement of the printing head or stage, allows for obtaining precise 3D constructs also with a high level of complexity.^[^ ^12^ ^]^

Despite the clear advantages of MEW, the method is still limited by a small number of well‐processable polymers. A suitable material for MEW should have a melting point within the printer's working temperature, moderate melt viscosity, low conductivity, good mechanical properties post‐processing, and biocompatibility.^[^ ^13^ ^]^ Polycaprolactone (PCL), the gold standard material for MEW, is one of the few materials that meets all those requirements. Due to its limited wettability that impedes cell attachment and growth, printed scaffolds are often plasma‐treated.^[^ ^14^ ^]^ Other thermoplastic polymers used in MEW include polylactic acid,^[^ ^15^, ^16^ ^]^ poly(lactic‐co‐glycolic acid),^[^ ^17^ ^]^ polypropylene,^[^ ^18^ ^]^ and poly(vinylidene difluoride).^[^ ^19^ ^]^ To obtain more durable scaffolds with better cell attachment and specific physicochemical properties, recently, a number of studies investigated modified polymers and block copolymers.^[^ ^19^, ^20^, ^21^ ^]^ For instance, Kade et al. investigated the MEW of poly(vinylidene fluoride‐co‐trifluoro ethylene) to obtain scaffolds with piezoelectric properties. However, due to the high viscosity of the polymer melt at the printing temperature, MEW was only possible at extremely low speed and high pressure. Also, non‐optimal attachment between fibers required the additional use of a heated collector.^[^ ^22^ ^]^ Interestingly, Sanchez Diaz et al. used poly(L‐lactide‐co‐ε‐caprolactone) to fabricate melt electrowritten scaffolds with high elasticity.^[^ ^23^ ^]^ To be able to effectively print at 110 °C, the authors pre‐degraded the polymer at 150 °C which facilitated extrusion.

Thermoplastic elastomers are interesting materials for MEW as they possess thermoplastic processability and elastomers' softness, and high extensibility in the elastic region.^[^ ^24^ ^]^These polymers are composed of different immiscible hard and soft segments that phase‐separate on the microscale. The hard segment, i.e., with low glass transition temperature, forms glassy domains that act as physical cross–links. While chemical cross–linking provides an irreversible bonding between polymer chains, physical cross–links provide reversible temperature‐dependent network rearrangement. This effect accounts for the suitability of the thermoplastic elastomers for various polymer melt processing techniques.^[^ ^25^ ^]^ One class of interesting thermoplastic elastomers used in tissue engineering are poly(ethylene oxide terephthalate)‐poly(butylene terephthalate) (PEOT‐PBT) multi‐block copolymers, also known as PolyActive. These multi‐block copolymers’ soft segment (PEOT) is hydrophilic and can absorb water,^[^ ^26^, ^27^ ^]^ while the hard segment (PBT) is hydrophobic and semi‐crystalline, providing mechanical stiffness. By changing the ratio of hard and soft segments as well as the molecular weight of the starting polyethylene oxide chain, their properties such as mechanical features, water absorption capacity, biodegradability, and biological response can be tailored to the need.^[^ ^28^ ^]^ Additionally, studies showed that PEOT‐PBT is biocompatible in vitro and in vivo.^[^ ^29^, ^30^ ^]^

PEOT‐PBT was used before in additive manufacturing for tissue engineering purposes. Anan et al. used PEOT‐PBT to fabricate a human‐like tympanic membrane by combining FDM printing and solution electrospinning. The authors produced fiber diameters ranging from ≈100 to 200 µm using the FDM printer and ≈0.5 to 1.8 µm using solution electrospinning. The obtained PEOT‐PBT constructs provided similar mechanical and acoustic features to the natural membrane. Biological studies revealed that both fibroblast and mesenchymal stromal cells can attach and grow on the fabricated scaffolds in a printed pattern‐dependent manner.^[^ ^31^ ^]^ In another study, Neves et al. used PEOT‐PBT in FDM printing of square‐mesh scaffolds with the lowest average fiber diameter of 69.4 ± 6.1 µm and evaluated the influence of single fiber surface topography on mesenchymal stromal cell activity. Under induction medium conditions, scaffolds with lower surface roughness showed a positive effect on hMSCs proliferation, whereas chondrogenesis was favored by rougher surfaces.^[^ ^32^ ^]^

The thermal and thermo‐oxidative degradation of polymers during printing is also an important factor to be considered. High temperatures and long residence time make many polymers susceptible to degradation during the printing procedure.^[^ ^23^ ^]^ Studies on PCL indicate that thermal stability and degradation rates vary significantly with processing conditions.^[^ ^33^ ^]^ Investigations into cyclic and constant heating have shown that prolonged thermal exposure at elevated temperatures can reduce molecular weight due to chain scission, particularly through ester hydrolysis. This highlights the importance of a thorough thermal analysis for new polymers used in MEW.

Here, we investigated for the first time the use of an elastomeric multi‐block copolymer, PEOT‐PBT, for MEW. Due to the high temperatures applied in MEW, first, we analyzed the thermal stability of PEOT‐PBT. Next, we defined printing parameters suitable to obtain a stable scaffold, including applied voltage, needle‐to‐collector distance, printing speed, printing temperature, and printing pressure. Finally, we proposed two specific designs for PEOT‐PBT scaffolds, with distinct mechanical properties, to engineer soft tissue mimics. The cellular response to those scaffolds was compared to the gold‐standard PCL. The cell morphology, percentage of pore bridging, myofibroblast differentiation, and ECM production were studied with mouse embryonic fibroblast cells until 28 days of culture. These cells were chosen due to their inherent role in the natural wound healing process and ECM production. The obtained results showcased that elastomeric PEOT‐PBT material should be added to the limited list of polymers used in MEW, broadening the applications of this technology in tissue engineering.

## 2 Experimental Section

### 2.1 Melt Electrowriting

**Figure 1:**
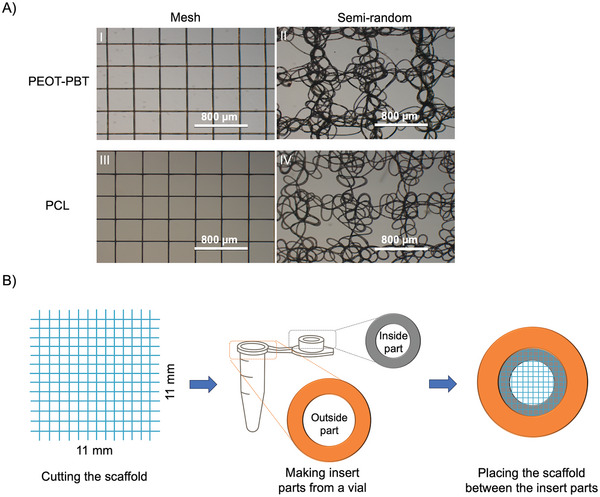
A) Inverted light microscopy images of melt electrowritten PEOT‐PBT mesh and semi‐random scaffolds (I and II), PCL mesh and semi‐random scaffolds (III and IV). B) Schematic illustration of insert preparation to fixate scaffolds for cell culture studies.

**Figure 2:**
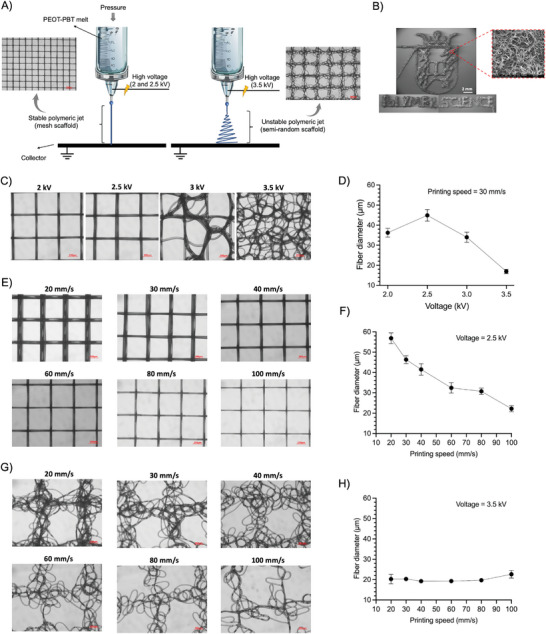
A) Increasing the voltage at a constant distance and printing speed can lead to jet instability and deposition of wavy fibers. B) SEM microscopy image of the University of Groningen and Polymer Science group logos printed with PEOT‐PBT at a voltage of 3.5 kV, printing speed of 20 mm s^−1^, pressure of 3 kPa, nozzle to collector distance of 2 mm, temperature of 195 °C, and nozzle diameter of 0.35 mm at different magnifications. Note that three images were stacked together for the low magnification image as the SEM device was not able to take an image from the whole printed logo. Here we showed that this printing condition can be used for applications that require printing complex structures with randomized micron‐size fibers. C) Light microscope images of PEOT‐PBT printed scaffolds at different applied voltages and printing speeds of 30 mm s^−1^ and D) their respective average fiber diameter. E) Light microscope images of PEOT‐PBT printed scaffolds at different printing speeds and voltage of 2.5 kV and F) their respective average fiber diameter. G) Light microscope images of PEOT‐PBT printed scaffolds at different printing speeds and voltage of 3.5 kV and H) their respective average fiber diameter. In all experiments, the pressure, nozzle‐to‐collector distance, printing temperature, and nozzle diameter were kept constant at 3 kPa, 2 mm, 195 °C, and 0.35 mm, respectively.

The PEOT‐PBT multi‐block copolymer with an initial polyethylen oxide length of 300 kDa and PEOT to PBT ratio of 55:45 was purchased from PolyVation, Netherlands. It was used to fabricate scaffolds with a melt electrowriting (MEW) printer (Spraybase, Ireland, and GESIM, Germany). For each printing session, the printer cartridge was filled with fresh PEOT‐PBT pellets and then flushed with nitrogen to remove the remaining air. Subsequently, the MEW printer was set at 195 °C and kept at that temperature for 30 min before printing in order to achieve a homogenous material melt. The adequate applied pressure, nozzle‐to‐collector distance, and nozzle diameter values were determined by some preliminary studies (data not shown). Then, the effect of different values of voltage (2 to 3.5 kV), and printing speed (20 to 100 mm s^−1^) on scaffold morphology was systematically studied. Based on those examinations, final printing parameters were chosen to print two different scaffold designs, mesh‐like and semi‐random structures, for mechanical testing and biological studies (**Table** **1** and **Figure** **1**A). The semi‐random scaffolds were composed of strands of random fibers deposited in an ordered manner (Figures 1A and **2**A,B). This structure was achieved by increasing the voltage while keeping the distance constant (Figure 2A). The formation of such a structure by controlling the voltage had already been reported by Bisht et. al.^[^ ^34^ ^]^ The designed strand‐to‐strand distance for mesh and semi‐random scaffolds were 400 and 800 µm, respectively. Note that for semi‐random scaffolds a single strand was composed of multiple separate fibers thus the actual pore gap become smaller than the designed strand‐to‐strand distance. For easy removal of the scaffolds from the collector stage after printing, 1–2 drops of ethanol were poured on the scaffolds, dried with gentle airflow, and then scaffolds were collected and kept in a dry place for further usage.

**Table 1:** Optimized parameters used to fabricate PEOT‐PBT and PCL mesh and semi‐random scaffolds for mechanical and cell culture studies. All scaffolds were printed with 8 layers (1 layer was understood here as fibers deposited by the printer in one, X or Y, direction).

Scaffold name	Designed pore gap	Printing temperature	Nozzle diameter	Printing pressure	Printing speed	Distance	Voltage
PCL Mesh	400 µm	100 °C	300 µm	25 kPa	13 mm s^−1^	3 mm	6.15 kV
PCL semi‐random	800 µm	100 °C	300 µm	20 kPa	3 mm s^−1^	3 mm	7.15 kV
PEOT‐PBT Mesh	400 µm	195 °C	250 µm	5 kPa	60 mm s^−1^	2 mm	2 kV
PEOT‐PBT semi‐random	800 µm	195 °C	350 µm	4 kPa	20 mm s^−1^	2 mm	3.5 kV

The PCL mesh and semi‐random scaffolds were fabricated as a control; printing parameters were included in Table 1.

### 2.2 Morphological Assessment

The morphology of printed scaffolds was investigated by an Inverted Phase Contrast Microscope (Axiovert 25, Zeiss). For mesh scaffolds, the mean fiber diameter was calculated by measuring 20 different fibers from three printed samples with ImageJ software (version 1.52q, National Institutes of Health). For semi‐random scaffolds, mean fiber diameter and gap distance were calculated by analyzing 50 fibers and 20 gaps from three printed scaffolds, respectively. Moreover, scanning electron microscopy (SEM) (FEI Quanta 400 FEG) was used to investigate scaffolds’ morphology. For this means, scaffolds were first coated with a gold sputter device (JEOL JFC‐1300) at 20 mA for 45 seconds and then SEM images were taken under high vacuum conditions at 3 kV or 10 kV accelerating voltage. Secondary electrons (using the Everhart‐Thornley Detector – ETD) and backscattered electrons (using the Solid State Detector – SSD) were detected.

### 2.3 Mechanical Characterization

The effect of different scaffold geometries (mesh and semi‐random) and post‐printing annealing on ultimate tensile strength, elongation at break, and Young's modulus was investigated by a universal testing machine (Instron 5565) using a 5N load cell in tensile mode. PEOT‐PBT and PCL scaffolds were printed at 195 and 100 °C, respectively, using optimized printing parameters (see Table 1 for details on the parameters used). Then, the scaffolds were cut into 10 × 30 mm pieces, and placed between the device clamps, and tensile tests were performed at a constant pulling rate of 6 mm min^−1^ until the sample broke or the machine's limit was reached. The scaffold thickness was measured using a micrometer screw gauge. The effective cross‐section area was calculated by multiplying the scaffold's thickness by its width. The elastic regions were determined using linear regression, confirming that the *R* ^2^ value was >0.9. The Young's modulus was then determined from the slope of the linear region. To calculate the yield strain, another line was plotted with the slope of the linear region and a 0.2% strain offset. Yield strain was defined as the x‐coordinate of the point where the 0.2% offset line intersected the stress‐strain curve.^[^ ^35^ ^]^

### 2.4 Thermogravimetric Analysis

To study the thermal degradation temperature of the PEOT‐PBT polymer at different atmospheric conditions, thermogravimetric analysis (TGA) was performed with TGA 5500 (TA Instruments). For this purpose, the mass loss of neat PEOT‐PBT polymer in air and nitrogen atmosphere was recorded from 28 °C to 500 °C with a heating rate of 10 °C min^−1^. Additionally, to assess the influence of heating time and atmospheric conditions on PEOT‐PBT degradation, an isothermal TGA analysis was conducted at 195 °C in air and nitrogen atmosphere. For this analysis, samples were initially heated from 20 °C to 195 °C at a rate of 20 °C min^−1^, followed by an isothermal step at 195 °C for 6 h.

### 2.5 Differential Scanning Calorimetry

Differential scanning calorimetry (DSC) (DSC Q1000, TA Instrument, USA) was performed on neat PEOT‐PBT and PEOT‐PBT samples that were heat‐treated at 195 °C for 6 h in nitrogen or air atmosphere to investigate the effect of these prolonged heating on PEOT‐PBT melting temperature (*T*_m_), crystallization temperature (*T*_c_), enthalpy of melting (Δ*H*_m_), and crystallinity degree (*w*_c_). Temperature scans were recorded from a heating, cooling, and heating cycle with a heating rate of 10 °C min^−1^ and 2 min equilibration at 40 and 200 °C. All DSC measurements were performed in nitrogen gas. The thermal characteristics of the samples (*T*_m_, *T*_c_, and Δ*H*_m_) were calculated by the Universal Analysis software (TA Instrument, USA) from the first cooling and second heating cycles. Moreover, the *w*_c_ of the samples was calculated using the following equation:

(1) $$w_{c}=\Delta H_{m}/\Delta H^{0}$$

Δ*H*_m_ was calculated from the heat fusion of samples during the second heating cycle. Δ*H* ^0^ was reported to be 144.5 J g^−1^ for PBT and thus 65.025 for PEOT‐PBT with a PBT ratio of 45%.^[^ ^36^ ^]^

### 2.6 Gel Permeation Chromatography

Gel permeation chromatography (GPC) was implemented to study the degradation of PEOT‐PBT during the printing process. The measurement was performed on a GPC Max system from Viscotek equipped with a refractive index detector and two columns in series (PLgel 5 µm MIXED‐C 300 mm from Agilent Technologies). The columns and detectors were maintained at a temperature of 35 °C. Chloroform (HPLC grade, amylene‐stabilized from Sigma‐Aldrich) was used as an eluent at a flow rate of 0.5 mL mi^−1^n. Near monodisperse polystyrene standards (M*n =* 645‐ 3 001 000 Da from Polymer Laboratories) were used for the construction of a calibration curve. Neat and heat‐treated samples (6 h at 195 °C under nitrogen or air atmosphere) were dissolved in the eluent at a concentration of ≈ 2 g/L and passed through a 0.45 µm PTFE filter prior to injection. Data acquisition and calculations were performed using Viscotek Omnisec software version 5.0. GPC elugrams were normalized with respect to the maximum of the polymer peak for better comparison of the series. Finally, the normalized refractive index was calculated and plotted as a function of the retention volume.

### 2.7 Proton Nuclear Magnetic Resonance Spectroscopy

To further investigate the thermal decomposition of PEOT‐PBT polymer during the printing process, proton nuclear magnetic resonance spectroscopy (^1^H‐NMR) was performed using a 400 MHz NMR machine (Avance III HD, Bruker). The neat PEOT‐PBT polymer and heat‐treated samples (6 h at 195 °C under nitrogen or air atmosphere) were dissolved in deuterated CHCl_3_ (Sigma‐Aldrich) at a concentration of ≈ 20 mg mL^−1^ and ^1^H‐NMR spectra were recorded using 16 scans.

### 2.8 Melt Rheology

The rheological characteristics of the PEOT‐PBT polymer melts were investigated using a rotational rheometer (HR‐2, TA Instrument) equipped with a forced convection oven fed with either nitrogen gas or air. 25 mm diameter stainless steel parallel plates were used for all the experiments. PEOT‐PBT polymers were hot pressed at 160 °C and 45 kN in a mold for 5 min to obtain circular disks. After loading the sample in the measuring area, a dynamic strain amplitude sweep was performed in order to determine the linear viscoelastic (LVE) regime, at an angular frequency of 100 rad s^−1^. Subsequently, a dynamic frequency sweep measurement was performed at frequencies ranging from 0.1 to 100 rad s^−1^ and a constant strain of 10%, within the LVE. The storage modulus (*G*′), loss modulus (*G*″), and complex viscosity (𝜂*) were plotted against angular frequency. Additionally, to have an insight into the rheological properties of PEOT‐PBT polymer during the printing process, a dynamic time sweep was performed at 1 rad s^−1^ frequency, and 10% strain in air and nitrogen atmosphere over 6 h. The *G*′, *G*″, and the 𝜂* were plotted as a function of time. All experiments were performed at 195 °C.

### 2.9 Water Contact Angle Determination Using In Situ ESEM

An FEI Quanta 400 FEG was used to determine the water‐wetting angle of individual fibers on the top of the scaffold in situ.^[^ ^37^ ^]^ Scaffolds were cut into small rectangular pieces and mounted to an Al holder with a 60° inclined surface using heat‐conductive double‐sided carbon tape. A Peltier cooling stage was installed into the ESEM and tilted to 30°. As a result, the surface of the scaffold was orientated 90° to the incoming electron beam (Figure S5A, Supporting Information). To perform in situ water wetting experiments the sample was cooled to 3 °C resulting in an equilibrium water vapor pressure of 750 Pa according to the P–T phase diagram of water. The condensation of water droplets was initialized by increasing the water vapor pressure to 1200 Pa. The observed water droplets on the flat part of the fiber represent advancing contact angles, measured using ImageJ (6 individual measurements).

### 2.10 Cell Culture Studies

The biocompatibility of printed scaffolds and the effect of their architecture on cell behavior were investigated during 28 days of in vitro cell culture studies. For this purpose, mouse embryonic fibroblast cell line (NIH‐3T3) (passage 26) was pre‐cultured in high glucose Dulbecco's Modified Eagle Medium (Sigma‐Aldrich, Germany) with 1% Penicillin‐Streptomycin (Gibco, USA), and 10% fetal bovine serum (FBS) (Gibco, USA) at 37 °C, 90% humidity, and 5% CO_2_. After 80% confluence was reached, cells were used for the biological studies.

#### 2.10.1 Cell Culture on the Scaffolds

PEOT‐PBT mesh scaffolds with an average fiber diameter of 19.4 ± 2.9 µm and gap distance of 378.1 ± 9.4 µm and PEOT‐PBT semi‐random scaffolds with an average fiber diameter of 19.9 ± 2.1 µm and gap distance of 438.6 ± 80.6 µm were printed, as described in Section 2.1 (Figure 1A). Next, to improve fiber‐fiber adhesion and prevent delamination during handling and cell culture, samples were treated in an oven at 125 °C for 30 min. As a control, PCL mesh scaffolds with an average fiber diameter of 18.3 ± 1.9 µm and gap distance of 378.2 ± 8.2 µm and PCL semi‐random scaffolds with an average fiber diameter of 20.6 ± 1.9 µm and gap distance of 450.3 ± 107 µm were used (see Section 2.1, Figure 1A).

All scaffolds were cut into 11 × 11 mm pieces with a rectangular blade, and placed in custom‐made Eppendorf inserts for easy handling and to prevent rolling in the cell culture medium, as shown in Figure 1B. Next, the scaffolds were surface‐treated with atmospheric plasma (plasma cleaner, Yocto, Germany) for 1 minute to improve the cell attachment, and were placed in non‐adherent 24‐well plates (Sarstedt, Germany). Subsequently, the scaffolds were sterilized with 70% ethanol for 60 min followed by three washes with phosphate buffered saline (PBS) solution. Afterward, scaffolds were incubated in a complete medium (see Section 2.9) for 90 min. For all the scaffolds, a seeding density of 1 × 10^5^ cells per cm^2^ on the scaffold was used. Cells seeded on glass coverslips in non‐adherent well plates at the density of 5000 cells per cm^2^ were used as controls (*n =* 3) for live–dead assay and Alamar blue assays (described below). The scaffolds were removed from their culture well and placed into a new well plate prior to the assays being performed.

#### 2.10.2 Alamar Blue Cell Metabolic Assay

To study the cell metabolic activity on MEW scaffolds (*n =* 3), the Alamar blue assay was performed according to the manufacturer's protocol (Invitrogen, Australia). Briefly, 1, 3, 7, 14, 21, and 28 days after cell culture, the culture medium was replaced with 1 mL of fresh medium containing 10% Alamar blue solution and scaffolds were incubated at 37 °C for 3 h (same 3 scaffolds were used at all the timepoints). 200 µl were taken from each sample, pipetted into a 96 well‐plate, and fluorescence values were measured with a spectrofluorometer (Varioskan Lux, Thermo Fisher Scientific, USA) at excitation and emission wavelengths of 560 and 590 nm, respectively. Finally, the cell metabolic activity was calculated by the following equation:

(2) $$\begin{array}{ccc} & & \mathrm{Alamar}\;\mathrm{blue}\;\mathrm{reduction}\%\\ & & =\frac{\mathrm{FI}\;590\;\mathrm{of}\;\mathrm{test}\;\mathrm{agent}-\mathrm{FI}\;590\;\mathrm{of}\;\mathrm{untreated}\;\mathrm{control}}{\mathrm{FI}\;590\;\mathrm{of}\;100\%\;\mathrm{reduced}\;\mathrm{alamarBlue}-\mathrm{FI}\;590\;\mathrm{untreated}\;\mathrm{control}}\\ & & \;\times \;100 \end{array}$$

where:FI 590 of the test agent was the fluorescence value obtained from scaffolds with cells.

FI 590 of 100% reduced Alamar blue fluorescence value obtained from autoclaved cell medium containing 10% Alamar blue solution. FI 590 of untreated control was the fluorescence value from the scaffolds without cells.

#### 2.10.3 Live–Dead Assay

To estimate the viability of cells seeded on the MEW scaffolds (*n =* 3), live–dead assay was conducted at different time points (days 1, 7, 14, and 28), as follows. Samples were stained with fluorescein diacetate (FDA) to observe living cells in green and with propidium iodide (PI) to observe dead cells in red. The staining solutions were prepared at concentrations of 5 µg mL^−1^ in PBS. First, the scaffolds were washed with PBS and then incubated with the staining solution for 10 min at 37 °C. After incubation, the scaffolds were rinsed twice with PBS and visualized under a confocal microscope (Olympus IX 81, Japan) using specific excitation/emission wavelengths for FDA (488/530 nm) and PI (561/620 nm). The living cells and dead cells were counted using the “find maxima” function in ImageJ software. The percentage of live and dead cells were calculated by using 3 images at 10x magnification covering an area of 0.486 cm^2^ from each sample.

#### 2.10.4 SEM Analysis

In order to further investigate cell morphology, cell–cell, and cell‐scaffold interaction, SEM imaging was performed after 1, 7, 14, and 28 days of culture. The scaffolds were washed two times for 5 min with PBS, afterward PBS was replaced with glutaraldehyde solution for storage till further use (2.5% v/v in 0.1 M sodium cacodylate buffer). The samples were stored for up to two weeks prior to imaging at 4 °C and further prepared just before imaging, by using a serial dilution method. In brief, scaffolds were first placed in 30% w/v EtOH aqueous solution and then moved to 50, 70, 80, 90, 96, 99, and 100% w/v EtOH aqueous solutions, consecutively. Scaffolds were kept at each step for 10 min before moving to the next step. Then, an equal amount of hexamethyldisilazane was added to the 100% w/v EtOH solution, before the scaffolds were placed two times in 100% w/v hexamethyldisilazane for 10 min. Finally, the hexamethyldisilazane was removed and the scaffolds slowly dried under ambient conditions. After careful drying, samples were sputter coated with gold (20 mA, 45 sec), and the SEM images were taken using the same protocol as described before.

#### 2.10.5 Immunofluorescence Staining

The immunostaining of the samples (*n =* 3) was done after 7, 14, and 28 days of culture. First, after 7 days of culture, vinculin staining for focal adhesions, phalloidin for cytoskeleton, and DAPI for nuclei were performed to assess the cell performance and cell‐scaffold interactions.

The staining process was performed as follows: scaffolds were washed once with PBS and fixed with 4% formaldehyde solution for 15 min, and stored at 4 °C until staining. For permeabilization, cell‐cultured scaffolds were treated with 0.1% Triton X‐100 in PBS (350 µL) for 10 min, followed by blocking with 1% Bovine Serum Albumin (BSA) in PBS (350 µL) for 15 min. Then the scaffolds were stained with Recombinant Alexa Fluor 647 Anti‐Vinculin Antibody (1:200 in PBS (350 µL), Abcam, UK) at 4 °C overnight, with Phalloidin iFLuor 488 (1:1000 in PBS (350 µL), Abcam, UK) at room temperature for 90 min, and with DAPI (2 µg mL^−1^ in PBS (350 µL) (Sigma, Germany) at room temperature for 10 min.

After 14 and 28 days of culture, Alpha smooth muscle actin (α‐SMA) and collagen type I antibody staining were performed to investigate the differentiation of fibroblasts to myofibroblasts and ECM production, respectively. Additionally, the samples were stained with DAPI and phalloidin, to visualize the cell nuclei and actin cytoskeleton.

The staining was performed as follows: scaffolds were washed once with PBS, then fixed with 4% formaldehyde solution for 10 min, and stored at 4 °C until further use. Next, for cell permeabilization, the scaffolds were treated with 0.1% Triton X‐100 in PBS (350 µL) for 15 min, followed by blocking with 1% Bovine Serum Albumin (BSA) in PBS (350 µL) for 15 min. Then, samples were stained with α‐SMA Monoclonal Antibody (1A4) eFluor 660 (1:500 in PBS (350 µL), Thermo Fisher Scientific, USA) and primary Rabbit Anti Mouse‐Collagen‐I Antibody (1:300 in PBS (350 µL), Abcam, UK) at 4 °C for overnight, followed by incubation in a secondary Goat Anti‐Rabbit IgG H&L Alexa Fluor 568 (1:500 in PBS (350 µL), Abcam, UK) and Phalloidin iFLuor 488 (1:1000 in PBS (350 µL), Abcam, UK) for 90 min, and finally DAPI staining (2 µg mL^−1^ in PBS (350 µL)) (Sigma, Germany) for 10 min at room temperature. All staining experiments were performed at dark conditions and the samples were washed three times with PBS after each step of the staining process. Finally, the scaffolds were placed on thin coverslips and visualized under an inverted confocal microscope (Olympus IX 81, Japan) from bottom to top. The same level of laser power (30%) and photo‐multiplier were used to observe the Collagen type I, α‐SMA, and vinculin expression. Whereas, to visualize the nuclei and actin expression, the same level of laser power (30%) was used but the photomultiplier was adjusted to obtain clear images.

### 2.11 Statistical Analysis

A one‐way ANOVA test with Tukey post‐hoc test was applied to the obtained data to determine statistical significance by using GraphPad Prism 8.0. Only for Alamar blue reduction assay two‐way ANOVA with Tukey post‐hoc test was applied. All the analyses were performed in triplicate experiments and mean ± standard deviation was reported. *P* values under 0.05 were considered statistically significant (*p < 0.05, **p < 0.01, ***p < 0.001, and ****p < 0.0001).

## 3 Results and Discussion

### 3.1 Printability

The effect of different MEW‐printing parameters and the overall printability of PEOT‐PBT were investigated. The first investigated parameter was the applied voltage, which is one of the most influential parameters in jet stability.^[^ ^38^ ^]^ While a minimum voltage value is required to form the polymer jet, increasing the voltages higher than a certain value can lead to jet instability. Here, the voltages of 2, 2.5, 3, and 3.5 kV were applied while other parameters were kept constant (printing speed = 30 mm s^−1^, pressure = 3 kPa, distance = 2 mm, and temperature = 195 °C). At a voltage below 2 kV, a continuous jet was not able to form due to inadequate pulling force (data not shown). At a voltage of 2 kV and 2.5 kV smooth mesh‐like scaffolds were obtained (Figure 2C). Further, a larger fiber diameter was observed at 2.5 kV (44.92 ± 2.88 µm) compared to 2 kV (36.25 ± 2.22 µm) (Figure 2D). We assign this effect to a higher pulling force at higher voltages leading to a higher flow rate and, consequently, larger fibers.^[^ ^34^ ^]^ By increasing the voltage to 3 kV and above, the polymer jet became unstable, causing a distortion in the morphology of the obtained scaffolds. Also, by increasing the voltage to 3 and 3.5 kV, the fiber diameter continuously decreased. At this voltage, the fibers go through a circular pattern before reaching the collector (whipping), which causes stretching into smaller diameters.^[^ ^39^, ^40^ ^]^

Next, the effect of printing speed was studied at the voltage of 2.5 and 3.5 kV. Results showed that PEOT‐PBT scaffolds could be successfully printed in a broad printing speed ranging from 20 to 100 mm s^−1^ (the device limit) at both investigated voltages (Figure 2E,F). Faster printing speed is generally more desirable as the scaffold production rate is higher.^[^ ^22^ ^]^ However, faster printing speed may also cause jet lag which disrupts accurate fiber deposition, especially near the scaffold's edges.^[^ ^41^ ^]^ At 2.5 kV, only above 60 mm s^−1^ jet lag started to be apparent near the edges (data not shown). At 2.5 kV, all the investigated speeds were below the critical translation speed (CTS‐ the minimal printing speed necessary to obtain straight fiber), while at 3.5 kV they were all above the CTS. As observed before, the results of our study also showed the significant effect of voltage on the CTS.^[^ ^42^ ^]^

At 2.5 kV, allowing a stable polymer jet and printing of mesh‐like scaffolds, increasing printing speed from 20 to 100 mm s^−1^ significantly reduced the fiber diameter from 56.9 ± 2.64 to 22.2 ± 1.47 µm (Figure 2F)‐ a trend that is reported before.^[^ ^43^ ^]^ However, at 3.5 kV (unstable polymer jet), the fiber diameter did not show any changes upon increasing printing speed (Figure 2H). At this voltage, the jet instabilities lead to whipping and fiber stretching before deposition on the collector (Figure 2A). As a result, pulling on the material due to the increased printing speed, resulted in a reduced density of deposited fibers instead of a decrease in the fiber diameter (Figure 2G).

### 3.2 Chemical and Thermal Characteristics of PEOT‐PBT Before and After Printing

**Figure 3:**
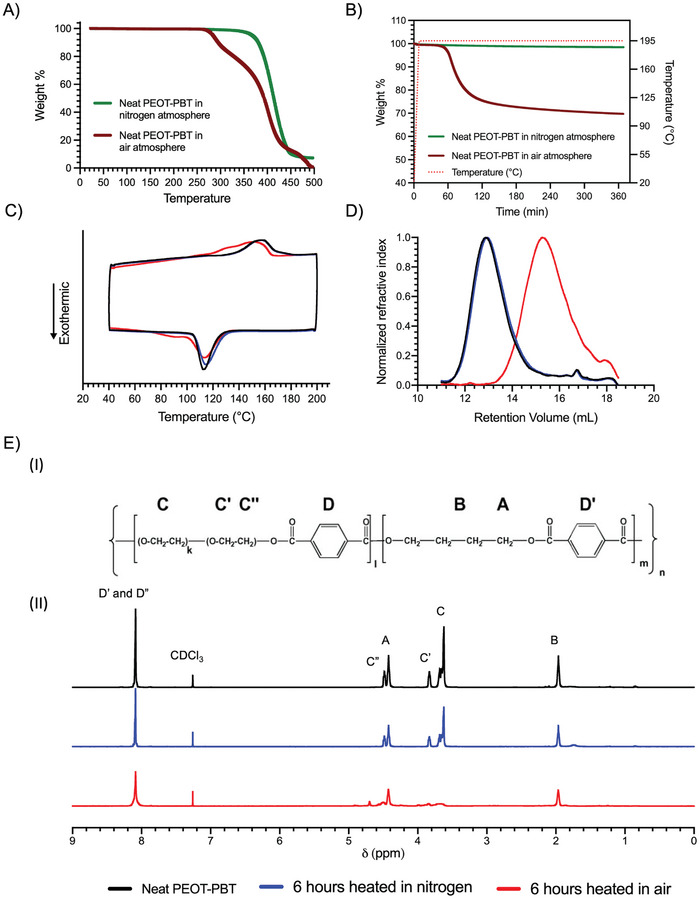
A) TGA of neat PEOT‐PBT in nitrogen and air atmosphere and B) isothermal TGA at 195 °C in air and nitrogen atmosphere. C) DSC, D) GPC, and E) I) chemical structure of PEOT‐PBT and II) ^1^H‐NMR analysis of neat, heat‐treated in nitrogen, and heat‐treated in air PEOT‐PBT.

In order to analyze if prolonged printing at high temperatures has a detrimental influence on the printed material, we compared the properties of PEOT‐PBT before and after heating. The TGA results showed that the neat PEOT‐PBT started a sharp weight loss at ≈350 °C in a nitrogen atmosphere (**Figure** **3**A). However, neat PEOT‐PBT in an air atmosphere started a slow weight loss already at ≈265 °C, followed by a sharp weight loss at ≈370 °C. This shows that the presence of oxygen reduces the thermal stability of PEOT‐PBT. This is due to a thermo‐oxidative degradation process that leads to chain scission at weak ester linkages. Moreover, the isothermal TGA results at 195 °C showed that neat PEOT‐PBT in a nitrogen atmosphere only lost 1.5% of its original weight during 6 h of heating (Figure 3B). On the other hand, neat PEOT‐PBT in an air atmosphere showed a drop in its original weight starting 45 min after heating. In air, PEOT‐PBT lost 30% of its original weight after 6 h of heating. These results showed that PEOT‐PBT at 195 °C in a nitrogen atmosphere is thermally stable while it degrades drastically in an air atmosphere.

Furthermore, the neat and heat‐treated PEOT‐PBT samples were investigated by DSC (Figure 3C). The neat and heat‐treated in nitrogen PEOT‐PBT samples showed broad endothermic peaks related to the melting temperature at ≈157.5 °C. This result is consistent with previous studies.^[^ ^25^ ^]^ Interestingly, the heat‐treated sample in the air showed a much broader endothermic peak with a melting point of 150 °C. The lower melting point and broader endothermic peak could be a result of PEOT‐PBT chain scission and the formation of new crystalline phases that crystalize at lower temperatures.^[^ ^44^, ^45^, ^46^ ^]^ Our crystallinity calculations showed that the neat and heat‐treated in nitrogen PEOT‐PBT samples have a crystallinity degree of 27.99 ± 0.31% and 27.35 ± 0.65%, respectively (Table S2, Supporting Information). These results proved that heat‐treating in nitrogen does not change the crystallinity degree of PEOT‐PBT. Finally, the heat‐treated in air PEOT‐PBT samples showed a much higher crystallinity degree of 42.23 ± 1.62%. The increase of crystallinity upon heating in the air atmosphere could be attributed to the thermo‐oxidative degradation of PEOT‐PBT, leading to an increase in the PBT crystalline portion.

To further examine the degradation, GPC analysis has been implemented. The change in retention volume indicated a substantial decrease (93.7%) in molecular weight when PEOT‐PBT was heated in the air environment. In turn, PEOT‐PBT heated in nitrogen showed only a slight decrease (9.8%) (Figure 3D; Table S1, Supporting Information). These results are consistent with the previous reports showing that thermo‐oxidative degradation is the most prominent degradation mechanism at this temperature range for PEOT‐PBT.^[^ ^47^ ^]^

^1^H‐NMR analysis was also employed to investigate the impact of thermo‐oxidative degradation on PEOT‐PBT, especially the change in PEOT to PBT ratio. To achieve this, the ratio between the backbone protons of the PEOT segment (C) and the PBT segment (B) was calculated by integrating the corresponding protons (Figure 3E). These specific proton groups were chosen to assess the degradation of the PEOT segment in comparison to the relatively stable PBT segment at the temperature range studied.^[^ ^47^ ^]^ The integration of B protons served as the reference point. Notably, the integration values for the PEOT backbone proton in the pristine, heat‐treated in nitrogen, and heat‐treated in air PEOT‐PBT samples were determined to be 2.51, 2.49, and 0.6, respectively. This indicates that the PEOT content of PEOT‐PBT heat‐treated in nitrogen remained almost the same as the neat sample while PEOT‐PBT heat‐treated in air experienced a substantial decrease of 76%.

The thermal and chemical analysis on PEOT‐PBT samples heat‐treated in air and nitrogen in comparison to non‐treated samples clearly shows that the thermal stability of PEOT‐PBT reduces noticeably while treated in air, due to thermo‐oxidative degradation of PEOT segment, while it remains relatively stable in nitrogen atmosphere. The change in soft‐to‐hard segment ratio due to thermo‐oxidative degradation suggests potential alterations in the material's physical properties. However, to better understand the polymer's behavior upon heating, particularly its melt flow properties crucial for MEW, we conducted melt‐rheology experiments.

First, strain and frequency sweeps were performed to find the linear viscoelastic (LVE) regime and assess the linear rheological behavior of PEOT‐PBT at the printing temperature (195 °C), respectively (Figures S1 and S2, Supporting Information). Frequency sweep measurements showed that the complex viscosity of PEOT‐PBT at 195 °C remains constant and independent from the angular frequency (Figure S2, Supporting Information), reflecting Newtonian behavior. This is most probably due to the short polymer chains of PEOT‐PBT and the relatively large distance of printing temperature from the melting point. Moreover, the frequency sweeps performed in air and nitrogen atmosphere showed similar values. This is due to the short heating time during the frequency sweep measurements which was not long enough to induce degradation in the air atmosphere (Figure S2, Supporting Information).

**Figure 4:**
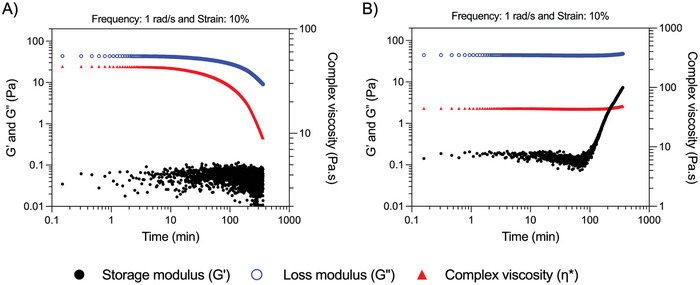
Dynamic time sweep measurement of PEOT‐PBT melt in A) air and B) nitrogen atmosphere. The temperature in both experiments was set to 195 °C.

Next, we performed dynamic time sweep analysis under air and nitrogen atmosphere (**Figure** **4**A,B) in order to investigate rheological changes of the PEOT‐PBT melt over time. The time sweep analysis of PEOT‐PBT melt under air atmosphere (Figure 4A) showed a drop in 𝜂* values after ≈10 min, showing a fast degradation upon heating in the presence of oxygen that adversely affects the viscosity and rheological behavior of the sample. Note that during rheology measurement the polymer melt was only exposed to air from the outer edges of the geometry. An increase in the contact area with air could have resulted in a more apparent drop in 𝜂* values. Interestingly, the time sweep analysis of PEOT‐PBT melt under a nitrogen atmosphere showed no change in *G*’ values up to 60 min after heating (Figure 4B), followed by an increase. Moreover, the 𝜂* stayed constant at ≈43 to 44 pa.s for ≈180 min and then start increasing slowly to reach 48 pa.s after 360 min. It is important to note that heat treatment in a nitrogen atmosphere did not change the polymer's crystallinity degree nor induce any chemical cross–linking as we showed before by DSC, GPC, and NMR measurements. We hypothesize that this increase in *G*’ and 𝜂* in time can be attributed to a micro‐phase separation of soft and hard segments and formation of physical cross–links. The micro‐phase separation and solidification above the melting point have already been reported for PEOT‐PBT.^[^ ^25^ ^]^ Vanzanella et. al showed that even above the melting point and below a critical temperature (known as the order‐disorder transition temperature), the repulsive forces between the hard and soft segments can overcome the Brownian motion over time, leading to the formation of the physical cross–links and solidification of this copolymer.

Altogether, our degradation studies indicate that the printing window for PEOT‐PBT in air and at 195 °C is very short and the material degrades upon exposure to oxygen and changes its properties. In a nitrogen atmosphere, it is evident that the material does not degrade. However, the rheological properties of PEOT‐PBT in nitrogen and at 195 °C can change over a long heating time, so it is better to keep the printing window short (≈2 h). It is also worth mentioning that in application, this can be compensated by changing the MEW chamber to a filament base system, with localized heating to avoid long exposure to heating.^[^ ^15^ ^]^

### 3.3 Mechanical Characterization

**Figure 5:**
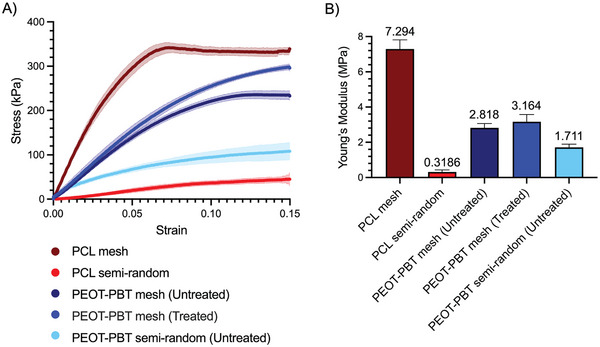
A) Tensile test results of untreated PEOT‐PBT mesh scaffolds, PCL mesh scaffolds, untreated PEOT‐PBT semi‐random scaffolds, PCL semi‐random scaffolds, and PEOT‐PBT scaffolds treated for 30 min at 125 °C. Figures represent an average of replicated experiments ± SD (the standard deviation is shown by a light color). Figures were cut at 0.15 strain for a better view of the elastic region. The full range of data is available in Figure S3 (Supporting Information). B) Average Young's modulus of different samples calculated from 6 replicates. All PEOT‐PBT and PCL mesh scaffolds have an average fiber diameter of ≈20 µm and a gap size of 400 µm.

The mechanical properties of scaffolds are among the most important factors determining the suitability of materials for tissue engineering and regenerative medicine applications.^[^ ^48^ ^]^ In our study, the stress‐strain curves for PEOT‐PBT mesh scaffolds displayed a wider linear elastic region than PCL, extending beyond 5.8% strain versus 3.4% for PCL (**Figure** **5**A,B). This finding suggests that PEOT‐PBT mesh scaffolds have a higher yield strain compared to PCL mesh scaffolds.

Moreover, PEOT‐PBT mesh scaffolds showed a more compliant nature with Young's modulus of 2.82 ± 0.25 MPa compared to stiffer PCL mesh scaffolds with a Young's modulus of 7.29 ± 0.52 MPa. Both materials in the semi‐random scaffold configuration exhibited lower Young's moduli compared to their mesh counterparts (Figure 5F). The deposition of material in semi‐random scaffolds results in coiled structures, reducing the effective Young's modulus. PEOT‐PBT semi‐random scaffolds showed higher Young's modulus (1.71 ± 0.18 MPa) compared to the PCL semi‐random scaffolds (0.32 ± 0.1 MPa) (Figure 5F). This might be due to higher fiber density in PEOT‐PBT semi‐random scaffolds than in the PCL ones (Figure 1A).

We also performed tensile tests on scaffolds that were heat‐treated for 30 min at 125 °C to see the effect of post‐annealing on mechanical properties. Results indicate that the average Young's modulus of the heat‐treated samples slightly increased compared to the non‐treated ones (by 0.346 MPa, see Figure 5F). This slight increase might be due to the fact that Young's modulus in the tensile test is not significantly influenced by the fiber‐fiber connection in the opposite direction of the stretching in mesh‐like scaffolds. We also observed that some treated samples failed relatively sooner compared to non‐treated ones (Figure S3, Supporting Information). We assign this effect to the partial sample degradation at higher temperatures in the presence of oxygen.

Our data are corroborated by previous research showing that PCL is stiffer than PEOT‐PBT, and PEOT‐PBT is more extensible.^[^ ^28^, ^49^ ^]^ The availability of well‐printable material with lower stiffness broadens the MEW application, as softer tissues can also be targeted.

### 3.4 Evaluation of MEW Scaffold's Biological Activity

PCL and PEOT‐PBT mesh and semi‐random scaffolds were tested for their biocompatibility and the cellular response specific to fibroblasts in tissue healing using NIH3T3 cells. To prevent fiber delamination and better handling, PEOT‐PBT scaffolds were heat‐treated in an oven at 125 for 30 min post‐printing. The light microscopy and SEM images of PEOT‐PBT scaffolds before and after heat‐treating can be found in Figure S4 (Supporting Information). The SEM analysis did not reveal any noticeable differences in the bonding at the fiber connection points after heat treatment. However, we observed that annealed scaffolds exhibited less delamination during the insertion process (Figure S4A,B, Supporting Information), suggesting that the annealing step improved the overall stability of the scaffold, even though visible fiber bonding changes were not detected. Moreover, our mechanical analysis showed an improved Young's modulus confirming this observation (Figure 5). Moreover, to improve cell attachment, air plasma treatment was introduced. In situ ESEM water wetting experiments showed that untreated fibers on PEOT‐PBT scaffolds have much lower water contact angle compared to untreated fibers on PCL scaffolds (42.3° ± 5.9° for PEOT‐PBT and 71.8° ± 3.1° for PCL). Results showed that the wettability of fibers on both PEOT‐PBT and PCL mesh scaffolds can be improved significantly by plasma treatment (Figure S5B, Supporting Information). After 30 sec of plasma treatment, the water contact angle of PEOT‐PBT and PCL mesh scaffold fibers decreased to 24.8° ± 4.2° and 31.4° ± 6.2°, respectively. The reason behind this significant wettability improvement is the addition of oxygen‐rich hydroxyl and carboxyl groups on the surface of plasma‐treated samples.^[^ ^50^, ^51^ ^]^ Note, that conventional set‐ups measure the water contact angle of a relatively large area which can be significantly influenced by the geometry.^[^ ^52^ ^]^ Considering the fiber diameter in this study (≈20 µm) and NIH3T3 cell size (≈15 µm), we assumed that cells would mostly be influenced by a single fiber's wettability rather than the overall scaffold's wettability. To deconvolute the effect of scaffolds' geometry on the surface wettability, here, we performed water contact angle measurement using ESEM.^[^ ^37^ ^]^ By this technique, we were able to measure the contact angle of single fibers, providing a better understanding of the wettability of the surface relevant to the cell attachment.

The cell culture experimental results showed a significant increase in cell attachment in plasma‐treated PEOT‐PBT mesh, PCL mesh, and semi‐random scaffolds compared to non‐treated scaffolds (Figure S6A, Supporting Information). In PEOT‐PBT semi‐random, we have noticed a positive trend of improved cell attachment on treated scaffolds (Figure S6A,B‐III,VII, Supporting Information). The improved cell attachment upon plasma treatment can be attributed to the increased wettability of the surface and the addition of polar groups.^[^ ^53^, ^54^, ^55^ ^]^ These findings are consistent with previous research by Cools et al., who observed improved cell adhesion and proliferation in PEOT‐PBT films after air, helium, argon, and nitrogen plasma treatments.^[^ ^51^ ^]^

After conducting preliminary assessments, we exclusively utilized MEW scaffolds treated with air plasma for subsequent biological experiments.

#### 3.4.1 Cell Viability

**Figure 6:**
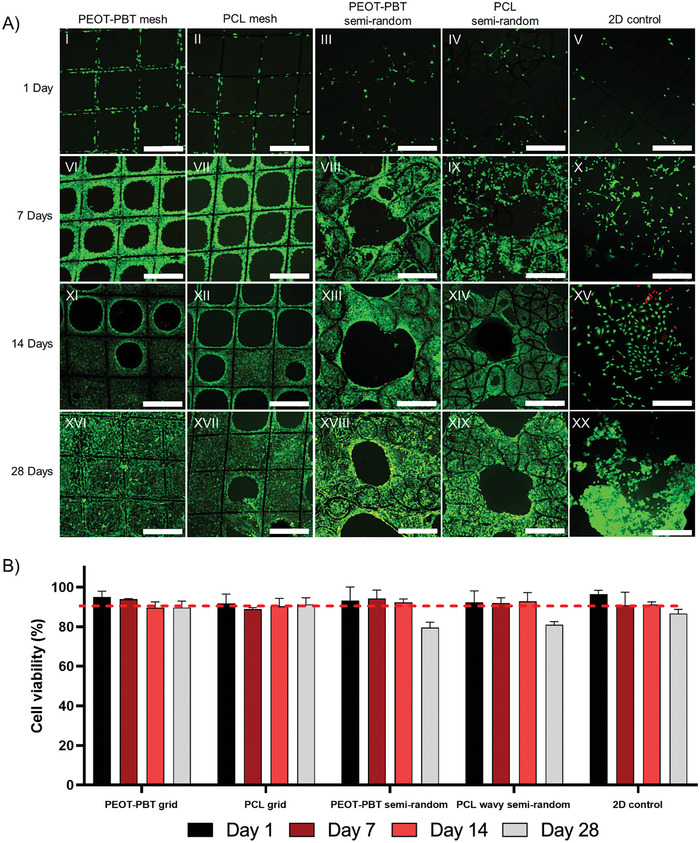
Representative live‐dead stained cells, imaged using confocal microscopy, of MEW scaffolds and 2D controls (*n =* 3) following 1 (I–V), 7 (VI–XI), 14 (XI–XV), and 28 (XVI–XX) days of NIH3T3 cell culture, respectively. Green indicates live cells and red indicates dead cells. Scale bar = 400 µm. B) Bar graph showing cell viability percentage calculated from live and dead cell images (*n =* 3) by ImageJ find maxima function on MEW scaffolds (*n =* 3).

The viability of the NIH3T3 cells was assessed with a live–dead assay after 1, 7, 14, and 28 days of culture. We observed a high number of live cells on all the scaffolds including 2D controls (stained in green), and only a few dead cells (stained with PI in red) (**Figure** **6**AI–XX). The cell viability remained consistently ≈90% during the 28‐day cell culture study, regardless of the material type and design used. However, a marginal decrease in cell viability (slightly below 90%) was observed on day 28 of culture for the semi‐random design, across both material types (Figure 6B). We attributed this effect to the local densely packed cell layers within the smaller pores of the scaffolds, which posed challenges in the effective exchange of oxygen and nutrients.^[^ ^56^ ^]^ Overall, the results suggested that both PEOT‐PBT and PCL MEW scaffolds offer favorable environments for cell growth without any toxic effects.

#### 3.4.2 Cell Metabolic Activity and Proliferation

**Figure 7:**
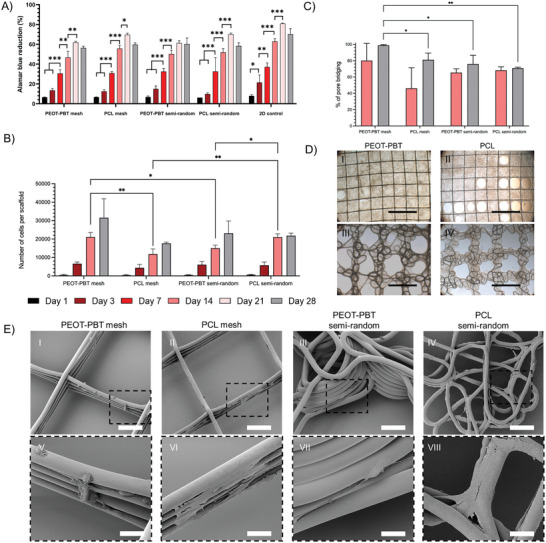
A) Bar graph representing Alamar blue reduction through cell metabolic activity on scaffolds and 2D controls (*n =* 3). B) Average number of cells per scaffold (*n =* 3) at each time point of culture calculated by nuclei counting with DAPI stained images using ImageJ find maxima function. C) Percentage of pore bridging calculated from low magnification brightfield images (*n =* 3). D) Representing bright field images after 28 days of culturing for pore bridging analysis (scale bar = 1000 µm). E) Scanning electron microscopy images of melt electrowritten scaffolds following 24 h culture (I to IV: scale bar = 100 µm and V to VIII: scale bar = 20 µm).

The Alamar blue reduction assay was employed to assess the metabolic activity of the cells. The increase in Alamar blue reduction indicates a higher level of activity. A significant increase in cell activity was seen from day 3 to day 21 in all the scaffolds and 2D controls (except for the PEOT‐PBT semi‐random scaffolds with no significant difference between day 14 and day 21) (**Figure** **7**A). The highest rate of cell metabolic activity was observed on day 21 across all the scaffolds, reaching a reduction of 61.95%, 69.6%, 61.2%, and 70% for PEOT‐PBT mesh, PCL mesh, PEOT‐PBT semi‐random, and PCL semi‐random scaffolds, including in 2D controls 79.5% respectively. However, no significant differences were observed between the scaffolds on day 21. The drop in cell metabolic activity on day 28 was observed for all the scaffolds and 2d controls, which can be attributed to reduced nutrient and oxygen exchange due to the high cell population.

To quantify the rate of cell proliferation, the number of cells on each scaffold at three random sites was counted using the DAPI‐stained samples. The number of cells increased in time on each scaffold type. However, after 21 days of culture (a trend also visible after 28 days), cell numbers were higher in the PEOT‐PBT mesh scaffolds than in the PCL mesh scaffolds (Figure 7B). This is also evident from the bright field microscopy (Figure 7C,D‐I,II). It was observed that after 28 days of culture, cells covered 99% of the PEOT‐PBT mesh scaffold's pores, whereas, at the same time point, only 80% of the PCL mesh scaffold pores were covered. In semi‐random scaffolds, PEOT‐PBT also demonstrated a higher pore bridging capacity (76%) compared to PCL (70%) (Figure 7C,D‐III,IV). The higher bridging rate of mesh scaffolds compared to the semi‐random scaffolds can be attributed to the larger pore gap between the main strands in the latter ones.

#### 3.4.3 Cell Morphological Study Through SEM Imaging

SEM imaging of NIH3T3 cells showed a distinct, typical fibroblast cell, spindle‐like morphology in mesh scaffolds after 1 day of culture (Figure 7E‐I,II,V,VI). The cells on semi‐random scaffolds were less elongated. This effect we assigned to the relatively small pores within the printed strands that could be bridged by the cells without clear elongation in one direction (Figure 7E‐III,IV,VII,VIII).

The differences not only between the designs but also between materials, were clearly visible. The majority of cells on PEOT‐PBT mesh and semi‐random scaffolds showed filopodia and lamellipodia structures with a rounded 3D cell shape after 1 day of culture (Figure 7E‐I,III,V,VII). In turn, a greater number of cells on PCL mesh and semi‐random scaffolds spread their cytoplasm flatly, covering the higher surface area on fibers (Figure 7E‐II,IV,VI,VIII). Similar cell behavior was seen on 2D controls grown on glass coverslips for 1 day (Figure S7B‐I,II, Supporting Information). This suggests that the cells on PEOT‐PBT scaffolds were actively migrating and extending filopodia to interact with their environment. In contrast, on PCL scaffolds, the cells preferred firm adhesion to the fibers.

After 7 and 14 days of culture, the cells were migrating and bridging the pores in circular patterns in all tested scaffolds. (Figure S7A, Supporting Information). In the longer term (i.e., cell culture up to 28 days), the cells seeded on PEOT‐PBT mesh scaffolds showed faster migration and bridging compared to PCL scaffolds (Figure S7A, Supporting Information). At 28 days of culture on PEOT‐PBT and PCL semi‐random scaffolds, the smaller pores of the strands were fully covered with cells but some of the larger pores separating the strands were still empty (Figures S7A‐III,IV,VII,VIII,XI,XII, Supporting Information).

#### 3.4.4 Immunofluorescence Analysis

Vinculin, actin, and DAPI stainings were employed to elucidate cell‐cell and cell‐fiber interactions within all scaffold types after the 7 days of culture. Regardless of the material type, mesh scaffolds exhibited organized vinculin adhesions in the fiber direction (Figure S8I‐I,II,V,IV, Supporting Information). For semi‐random scaffolds, vinculin adhesions were observed in all directions, indicating a diverse cellular response. These findings, in line with SEM observations, highlight the importance of scaffold architecture in shaping cell behavior and morphology. Similarly, organized cell morphology was observed on melt electrowritten PCL mesh scaffolds when cultured with neonatal human dermal fibroblasts (NHDFs), as confirmed by vinculin and actin staining.^[^ ^57^ ^]^

**Figure 8:**
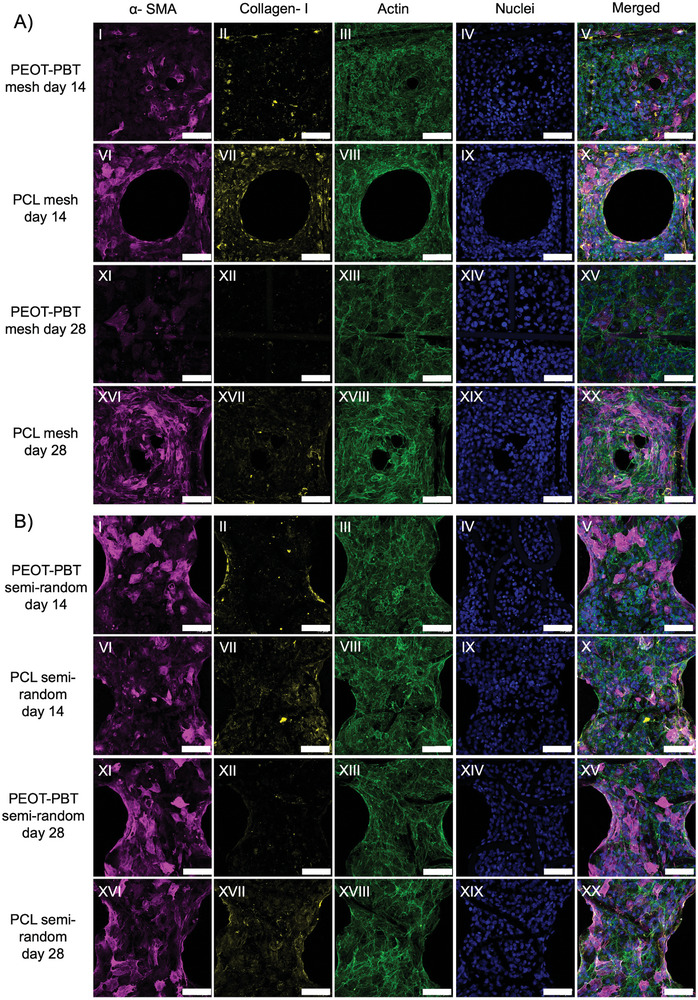
A) Immunofluorescence of α‐SMA (magenta), collagen I (yellow), actin (green), DAPI (blue) and merged in NIH3T3 cells after 14 days (I–X) and 28 days (XI–XX) of culture on mesh and B) after 14 days (I–X) and 28 days (XI–XX) of culture on mesh scaffolds(*n =* 3). B) Immunofluorescence of α‐SMA (magenta), collagen I (yellow), actin (green), DAPI (blue) and merged in NIH3T3 cells after 14 days (I–X) and 28 days (XI–XX) of culture on mesh and B) after 14 days (I–X) and 28 days (XI–XX) of culture on semi‐random scaffolds(*n =* 3). Scale bar = 50 µm.

Further, immunofluorescence was used to identify the differentiation of myofibroblasts and the expression of collagen type I during cell culture. After 14 days of culture on PEOT‐PBT and PCL mesh and semi‐random scaffolds, showed a higher number of cells with positive expression for α‐SMA in the form of cytoplasmic stress filaments compared to cells on PEOT‐PBT mesh scaffolds at 28 days (**Figure** **8**A‐I,VI,B‐I,VI). On the 28th day, very few cells expressing α‐SMA in the form of cytoplasmic stress filaments on PEOT‐PBT mesh scaffolds (Figure 8A‐XI), while the cells cultured on the PCL mesh and semi‐random and PEOT‐PBT semi‐random scaffolds showed maintained high expression of α‐SMA (Figure 8A‐XVI, B‐XI,XVI).

In correlation with α‐SMA activity, the expression of collagen type I was higher on the 14th day in all types of scaffolds (Figure 8A‐II,VII, B‐II,VII) compared to the day 28th in PEOT‐PBT mesh scaffold (Figure 8A‐XII). The expression of collagen type I was also higher for PEOT‐PBT and PCL semi‐random scaffolds and for the PCL mesh scaffold (Figure 8A‐XVII,B‐XII,XVII) when compared to the PEOT‐PBT mesh scaffold on day 28th (Figure 8A‐XII).

The high expression of myofibroblast marker α‐SMA and collagen type I in both scaffold types after 14 days suggests active involvement in bridging pores, possibly driven by cytoskeleton stress filament formation. Notably, in the case of PEOT‐PBT mesh scaffolds, this expression decreased after 28 days, likely due to the completion of pore bridging. In contrast, PCL mesh scaffolds continued to exhibit high expression of α‐SMA and collagen type I, possibly because pore bridging remained incomplete, promoting continued myofibroblast activation. Similarly, a recent study performed by Federici et al. showed the expression of α‐SMA marker on day 14 and day 28 of hMSCs cells cultured on the MEW scaffolds with 400 µm pore size.^[^ ^58^ ^]^ Another study showed that scaffolds with a pore size greater than 300 µm led to excessive cell proliferation, however, decreased differential gene expression and delayed or incomplete pore bridging compared to lower mesh size (≈200 µm).^[^ ^59^ ^]^ Based on our and others' reports, PCL scaffolds with pore size <400 µm are advantageous for promoting desired cell behavior.

Myofibroblasts are activated fibroblast phenotypes during tissue injury or damage. These cells upregulate alpha‐smooth muscle actin (α‐SMA), which is involved in forming stress fibers and contractile forces that help wound closure and promote tissue repair. During tissue healing, collagen type I production is also upregulated, mainly by myofibroblasts. Elevated levels of α‐SMA expression can lead to excessive deposition of ECM components, mostly collagen type I, that lead to fibrosis, which is the main cause of scar tissue formation or organ dysfunction.^[^ ^60^ ^]^ In a clinical study, researchers used compression‐molded PEOT/PBT scaffolds with 75% porosity and an average pore size of 182 µm as implants for mosaicplasty donor site filling. The study's findings revealed that these scaffolds facilitated the formation of fibrocartilage instead of fibrous tissue at the implanted surface.^[^ ^61^ ^]^ Our results may indicate that the PEOT‐PBT mesh scaffolds are less likely to lead to fibrosis during tissue regeneration. However, this hypothesis would have to be confirmed through long‐term in vivo studies.

The differences in cell behavior, proliferation, and differentiation observed on PEOT‐PBT and PCL scaffolds may be attributed to the scaffold's stiffness, which affects mechanotransduction.^[^ ^56^ ^]^ W. J. Hendrikson et al. 3D printed (using the Fused Deposition Modeling approach) PEOT‐PBT and PCL mesh scaffolds with fiber diameters of 186 ± 20 µm and 169 ± 9 µm, respectively. In the study, it was evident that PEOT‐PBT exhibited superior cell differentiation capabilities, particularly in promoting chondrogenic differentiation, and scaffolds displayed a lower friction coefficient, making it an ideal choice to guide cell proliferation, alignment, and tissue organization.^[^ ^62^ ^]^ In another study, authors explained that PCL scaffolds may lead to induced hypertrophy as they promoted the gene expression for cartilage destruction and ossification.^[^ ^63^ ^]^

The active migration and dynamic interaction of cells on PEOT‐PBT scaffolds, characterized by filopodia and lamellipodia structures, suggest that fibroblasts respond well to soft and elastic substrates, promoting faster cell proliferation and differentiation. On the other hand, the flattened cytoplasmic spread inhibitions and elevated α‐SMA expression in cells on stiffer PCL scaffolds suggest potential risks of prolonged myofibroblast activation, leading to fibrosis and scar tissue formation. Therefore, PCL mesh scaffolds may be more suitable for cell differentiation in situations where higher stiffness is required.^[^ ^64^ ^]^ Our finding suggests that melt electrowritten PEOT‐PBT scaffolds are an interesting alternative to PCL for soft tissue regeneration, with the possibility of further tailoring cell responses by design.

## 4 Conclusion

Here, we have successfully printed PEOT‐PBT using melt electrowriting (MEW) and provided various physicochemical characterizations of the printed material. Our research demonstrates that PEOT‐PBT printed scaffolds exhibit unique properties advantageous for soft tissue engineering applications, with a higher yield strain compared to traditional materials like PCL. By optimizing printing parameters, we were able to fabricate two distinct scaffold designs—mesh and semi‐random—with tailored mechanical and biological properties. The softer nature of PEOT‐PBT mesh scaffolds facilitated favorable conditions for fibroblast growth and proliferation while mitigating prolonged expression of α‐SMA. Conversely, the higher stiffness of PCL mesh scaffolds resulted in elevated and prolonged expressions of α‐SMA, indicating its potential utility in studying fibrotic tissue diseases. Overall, this study enriched the library of materials available for MEW with an elastomeric polymer, opening possibilities for new applications of the technology.

## Conflict of Interest

The authors declare no conflict of interest.

## Supporting information



Supporting Information

## Data Availability

The data that support the findings of this study are available from the corresponding author upon reasonable request.
