# Supplementary material for: Melt Electrowriting of Elastic Scaffolds Using PEOT‐PBT Multi‐block Copolymer
Source: Adv Healthc Mater. 2024 Dec 10;14(3):2402914. doi: 10.1002/adhm.202402914 (PMC11773127; doi:10.1002/adhm.202402914)
Supplement: Supplementary file 1 — Supporting Information [file ADHM-14-0-s001.pdf]

ADVANCED HEALTHCARE MATERIALS

Supporting Information

for *Adv. Healthcare Mater.*, DOI 10.1002/adhm.202402914

Melt Electrowriting of Elastic Scaffolds Using PEOT-PBT Multi-block Copolymer

*Armin Amirsadeghi, Pavan Kumar Reddy Gudeti, Sietse Tock, Marcus Koch, Daniele Parisi, Marleen Kamperman and Małgorzata Katarzyna Włodarczyk-Biegun**

Melt Electrowriting of Elastic Scaffolds using PEOT-PBT Multi-block Copolymer – Supporting Information

Armin Amirsadeghi [a, †], Pavan Kumar Reddy Gudeti [b, †], Sietse Tock [a], Marcus Koch [c], Daniele Parisi [d], Marleen Kamperman [a], Małgorzata Katarzyna Włodarczyk-Biegun [a, b, *]

[a] Polymer Science, Zernike Institute for Advanced Materials, University of Groningen, Nijenborgh 3, 9747 AG, Groningen, The Netherlands

[b] Biotechnology Centre, The Silesian University of Technology, B. Krzywoustego 8, 44-100, Gliwice, Poland

[c] INM - Leibniz Institute for New Materials, Campus D2 2, 66123 Saarbrücken, Germany

[d] Engineering and Technology Institute Groningen (ENTEG), University of Groningen, Nijenborgh 3, 9747 AG Groningen, The Netherlands

* Corresponding author

† Equal as the first author

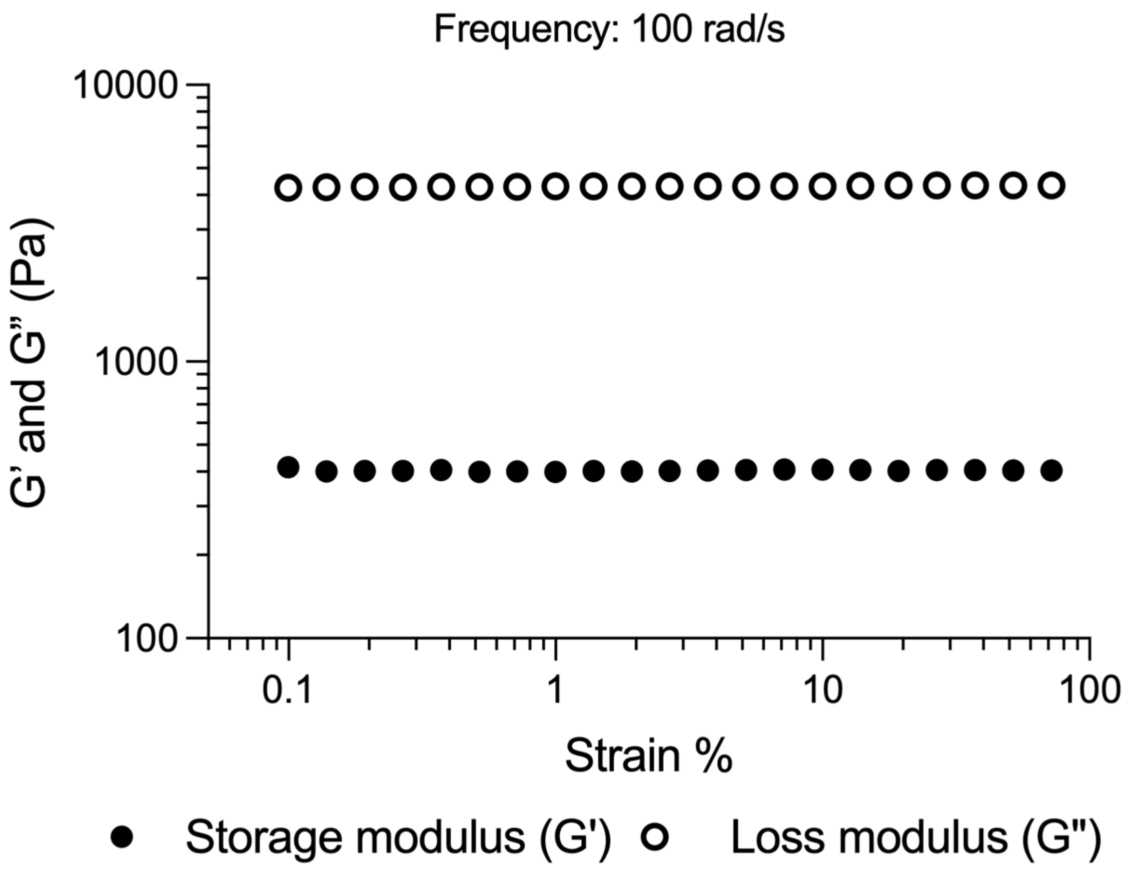

Figure S1. Strain sweep of PEOT-PBT at 195 °C from 0.1 to 100% strain and at 100 rad/s frequency in nitrogen atmosphere.

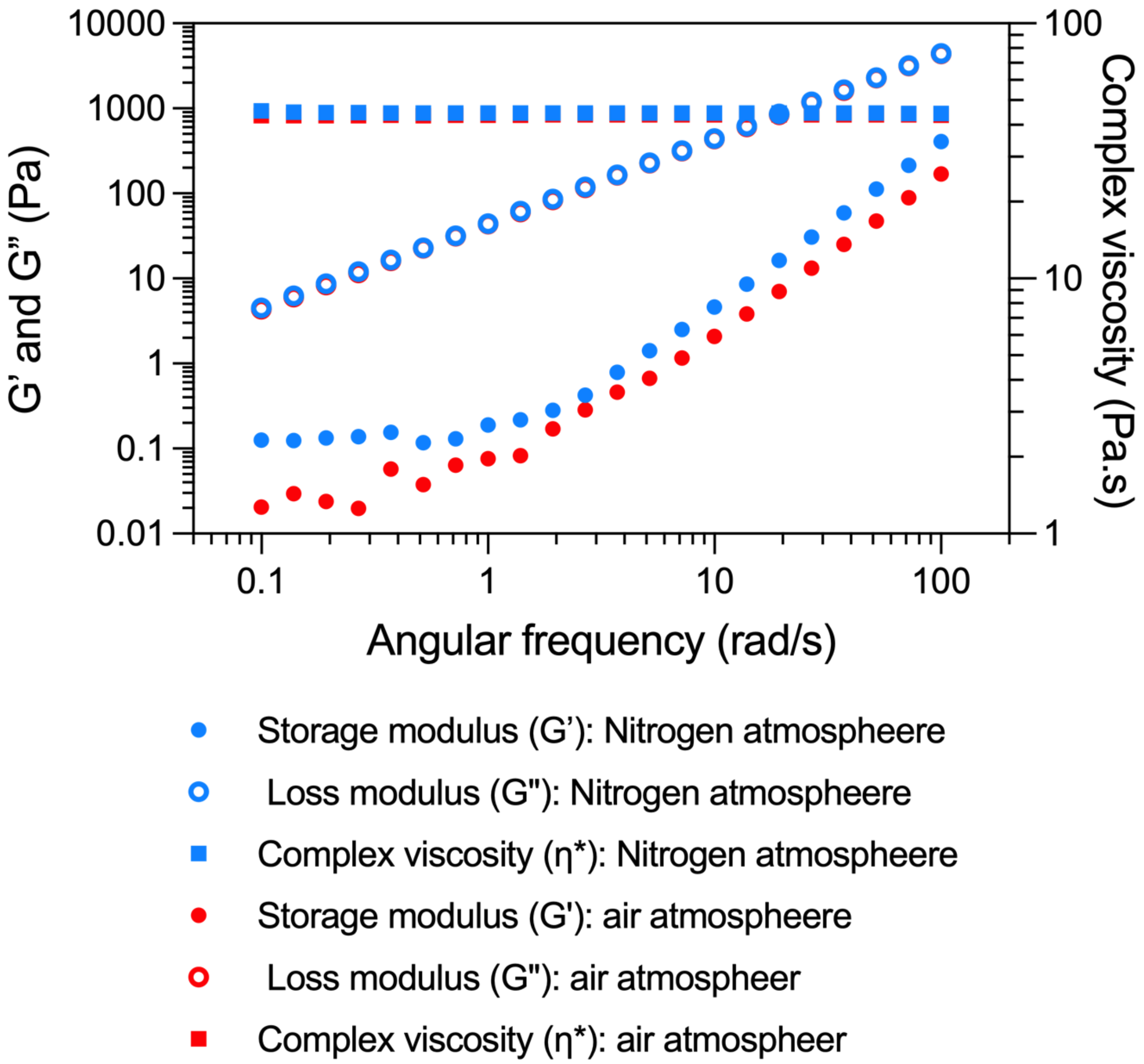

Figure S2. Frequency sweep of PEOT-PBT at 195 °C and 10% strain in air and nitrogen atmosphere.

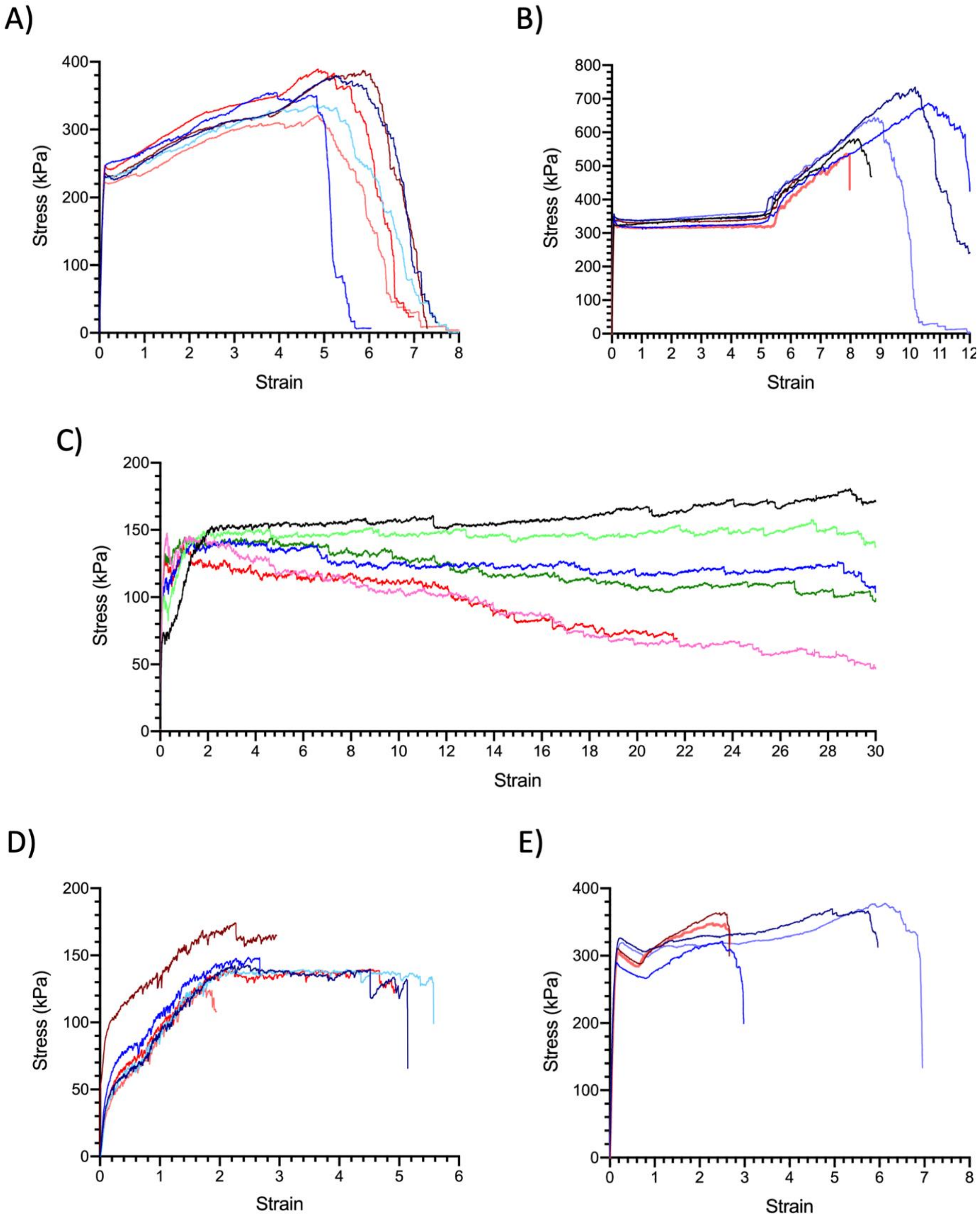

Figure S3. Tensile test results of A) untreated PEOT-PBT mesh scaffolds, B) PCL mesh scaffolds, C) untreated PEOT-PBT semi-random scaffolds, D) PCL semi-random scaffolds, and E) PEOT-PBT scaffolds treated for 30 minutes at 125 °C. All PEOT-PBT and PCL mesh scaffolds have an average fiber diameter of around 20 µm and a gap size of 400 µm.

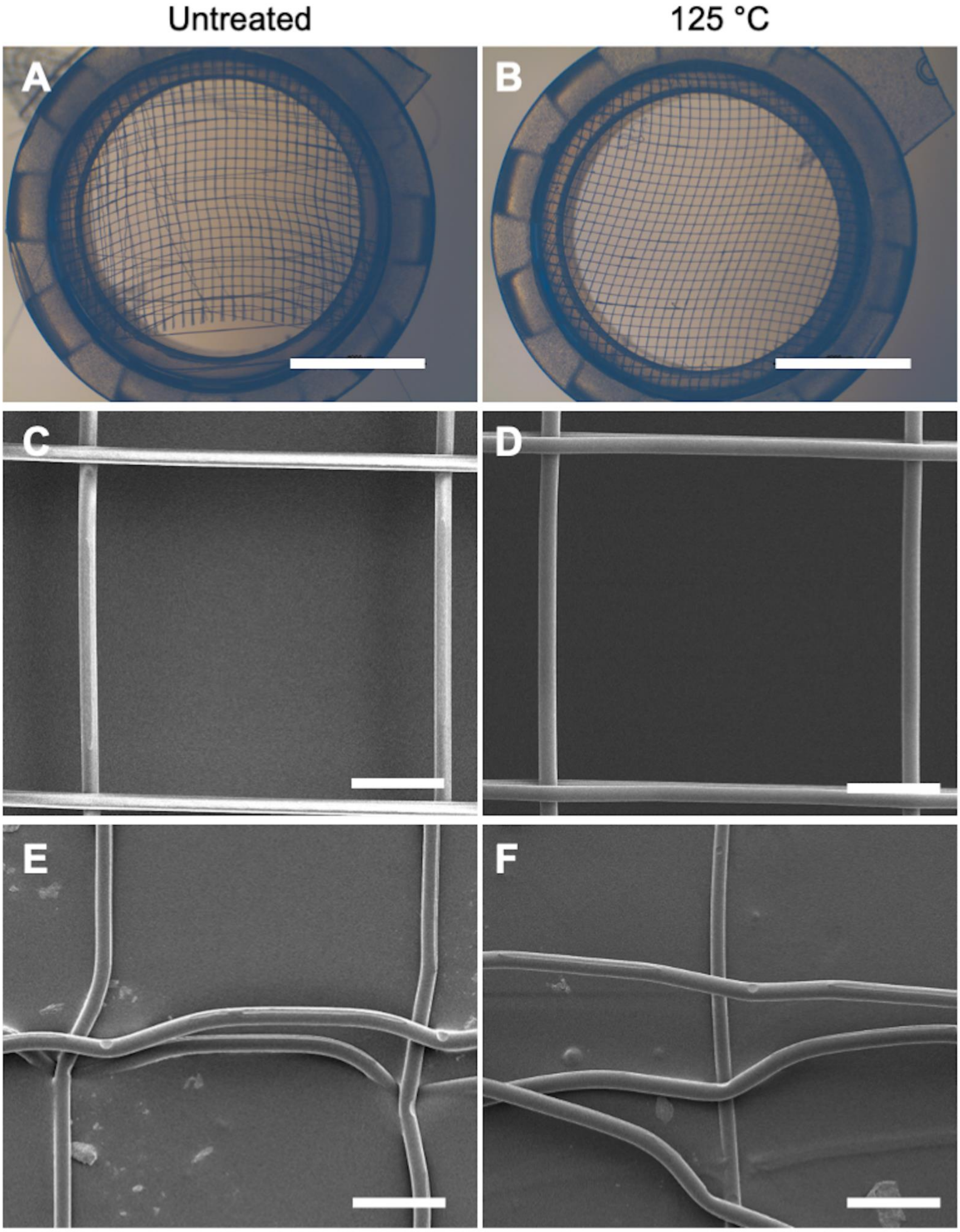

Figure S4. Microscope images of the A) untreated and B) heat-treated scaffold at 125 °C after the insertion process (scale bar = 2000 μm). SEM images showing the fiber morphology and connections of untreated (C and E) and heat-treated at 125 °C scaffolds (D and F) (scale bar = 100 μm).

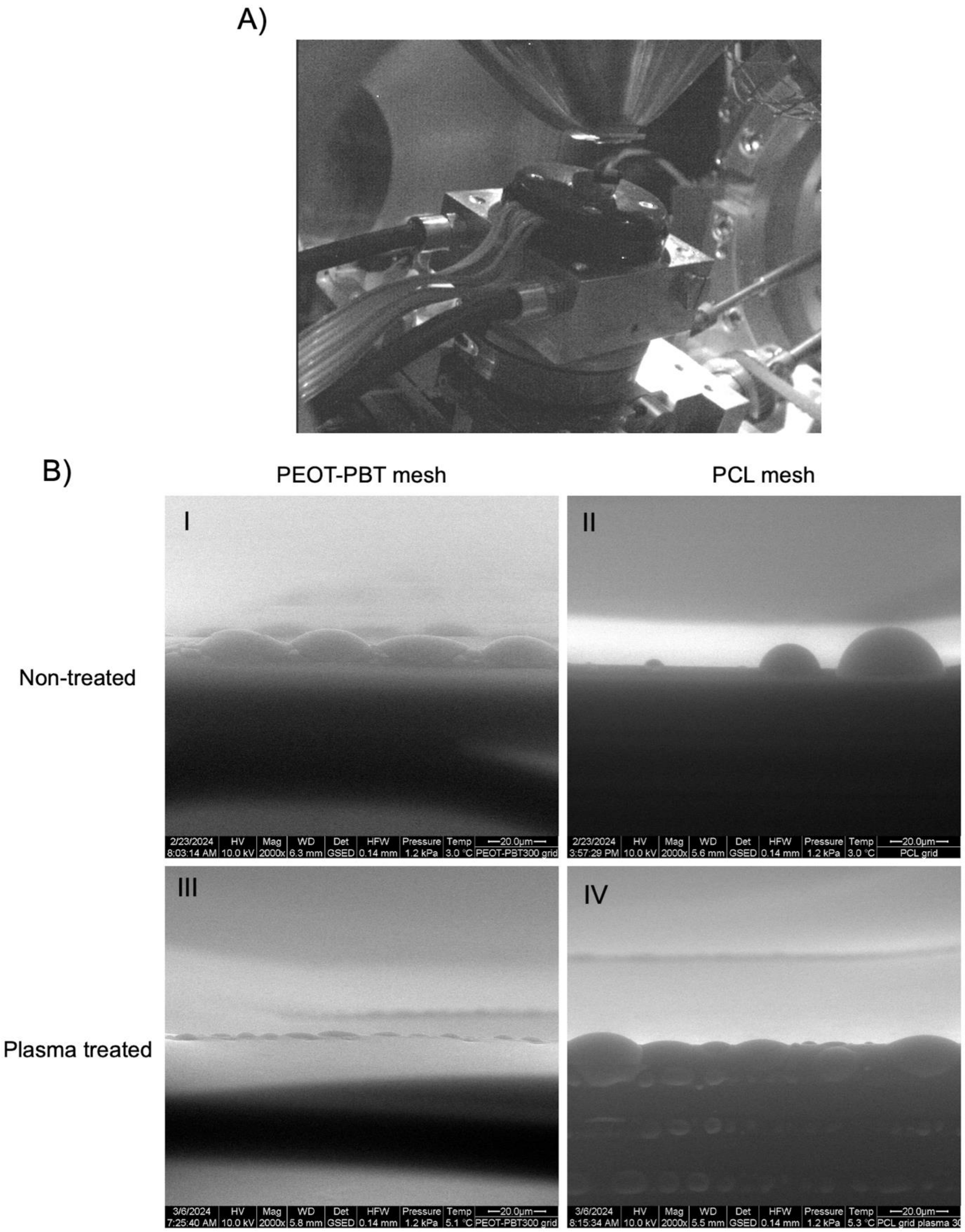

Figure S5. A) ESEM setup for in-situ water contact angle measurements. B) ESEM images of water droplets on PEOT-PBT and PCL mesh scaffolds before (I and II) and after (III and IV) plasma treatment.

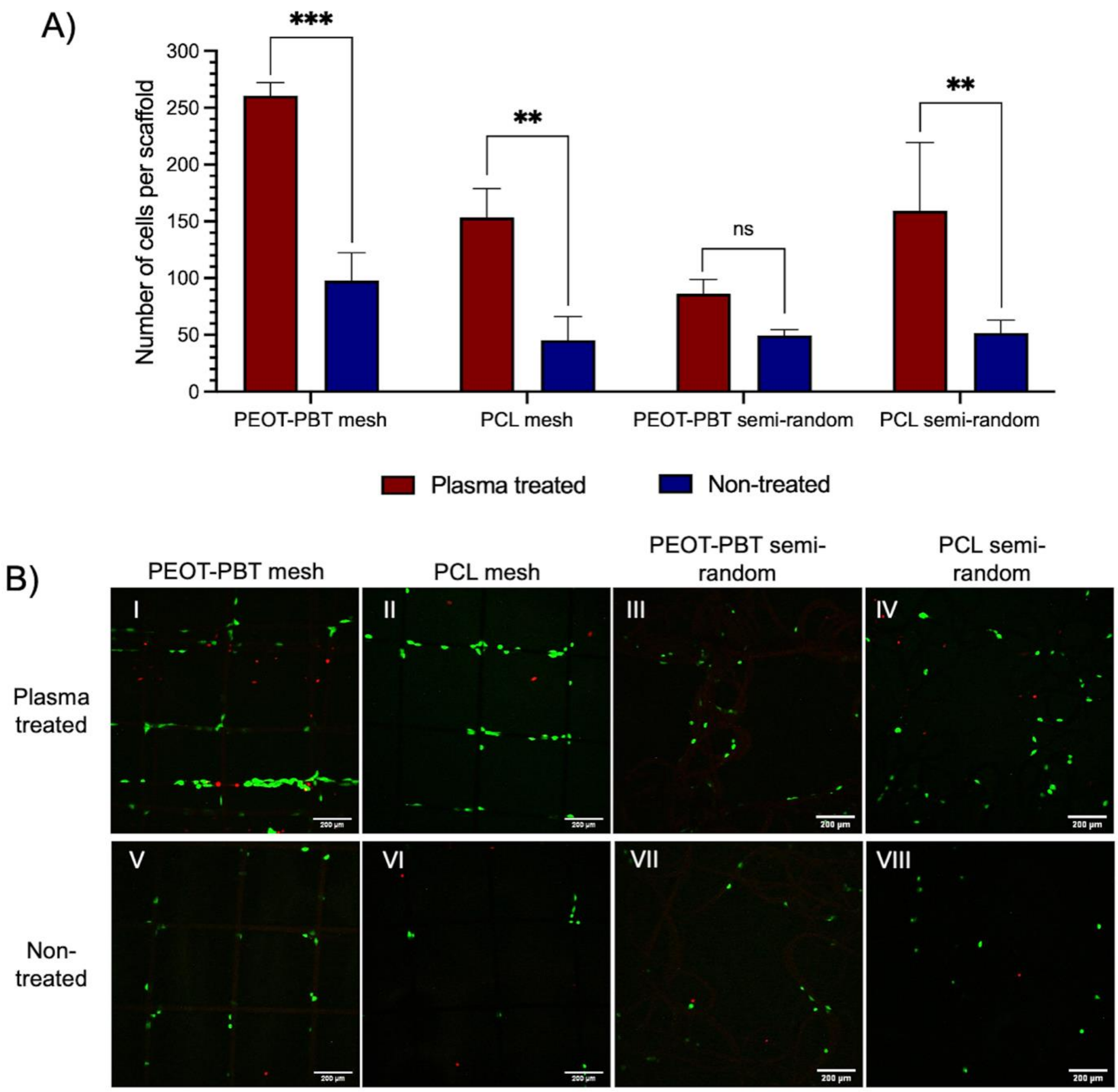

Figure S6. A) Bar graph showing cell viability percentage calculated from live and dead cell images by ImageJ find maxima function. B) Representative live-dead stained cells, imaged using confocal microscopy, of MEW scaffolds following 24 hours of NIH3T3 cell culture on plasma treated (I-IV) and non-treated (V-VIII), respectively. Green and red are indicative of live and dead cells, respectively. Scale bar = 200 µm.

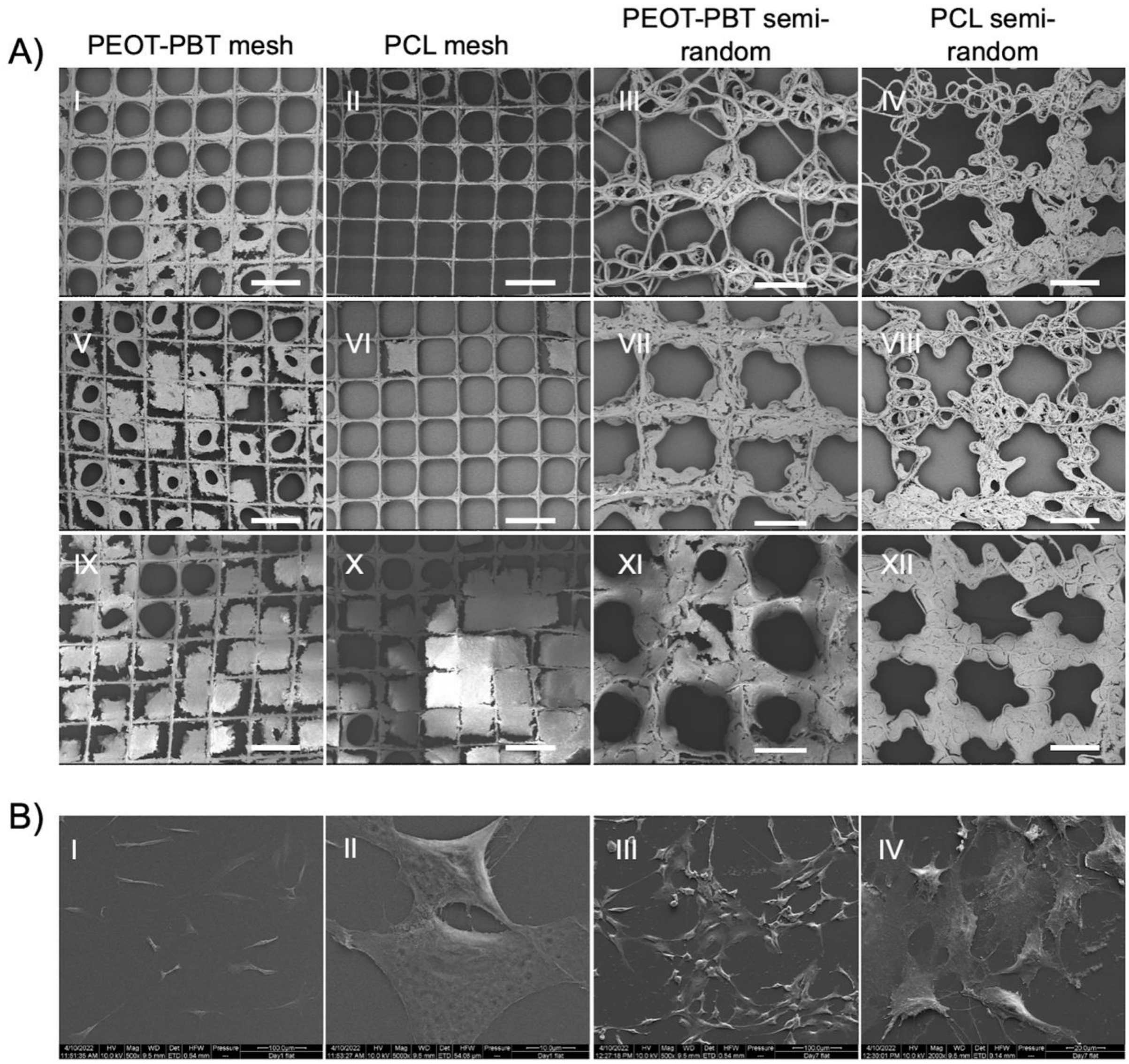

Figure S7: A) Representative SEM images of MEW scaffolds following 7 (I–IV), 14 (V–VIII), and 28 (IX-XII) days of NIH3T3 cell culture. Scale bar = 500 µm. B) SEM images of NIH3T3 cells grown on coverslips after 24 hours (I, II) and 7 days (III, IV).

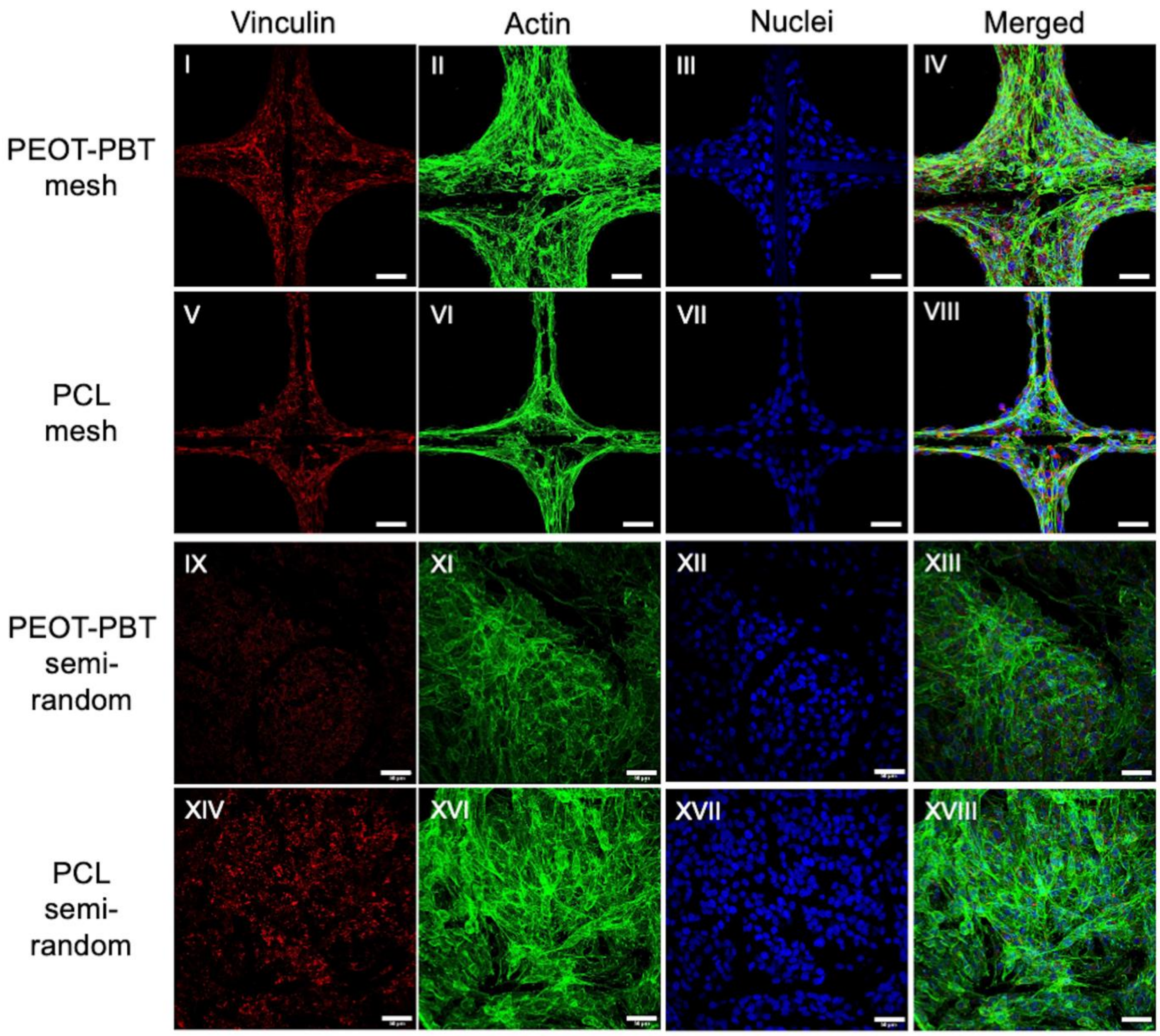

Figure S8. Immunocytochemistry of Vinculin (Red), F-actin (green) and DAPI (blue) in NIH3T3 cells after culture. Scale bar = 50 μm.

Table S1. GPC calculated weight average molecular weight (Mw) and number average molecular weight (Mn) of PEOT-PBT before and after heating at 195 °C in nitrogen and air.

**Sample**	**M_w (g mol^{-1})**	**M_n (g mol^{-1})**
**Neat PEOT-PBT**	59.03	30.14
**PEOT-PBT treated in nitrogen**	53.247	18.432
**PEOT-PBT treated in air**	3.734	658

Table S2. DSC calculated melting temperature (T_m), crystallization temperature (T_c), enthalpy of melting (ΔH_m) and crystallinity degree (w_c) of PEOT-PBT before and after heating at 195 °C in nitrogen and air from triplicate measurements.

**Sample**	T_m (°C)	T_c (°C)	ΔH_m (J/g)	w_c (%)
**Neat PEOT-PBT**	157.55 ± 0.28	113 ± 0.33	18.2 ± 0.2	27.99 ± 0.31
**PEOT-PBT treated in nitrogen**	157.48 ± 0.26	114.53 ±1.79	17.79 ± 0.43	27.35 ± 0.65
**PEOT-PBT treated in air**	150.67 ± 0.34	113.01 ± 0.52	27.46 ± 1.05	42.23 ± 1.62
